# Pre-stage perfusion and ultra-high seeding cell density in CHO fed-batch culture: a case study for process intensification guided by systems biotechnology

**DOI:** 10.1007/s00449-020-02337-1

**Published:** 2020-04-07

**Authors:** Lisa Stepper, Florian Alois Filser, Simon Fischer, Jochen Schaub, Ingo Gorr, Raphael Voges

**Affiliations:** Bioprocess Development Biologicals, Boehringer Ingelheim Pharma GmbH & Co. KG, Biberach an der Riß, Germany

**Keywords:** CHO, Process intensification, *N* − 1 perfusion, Concentrated fed-batch, Next-generation sequencing

## Abstract

Process intensification strategies are needed in the field of therapeutic protein production for higher productivities, lower cost of goods and improved facility utilization. This work describes an intensification approach, which connects a tangential-flow-filtration (TFF) based pre-stage perfusion process with a concentrated fed-batch production culture inoculated with an ultra-high seeding density (uHSD). This strategy shifted biomass production towards the pre-stage, reaching up to 45 × 10^6^ cells/mL in perfusion mode. Subsequently, production in the intensified fed-batch started immediately and the product titer was almost doubled (1.9-fold) in an equivalent runtime and with comparable product quality compared to low-seeded cultures. Driven by mechanistic modelling and next-generation sequencing (NGS) the process had been optimized by selecting the media composition in a way that minimized cellular adaptation between perfusion and production culture. As a main feature, lactate feeding was applied in the intensified approach to promote cell culture performance and process scalability was proven via transfer to pilot-scale i.e., 20 L pre-stage perfusion and 80 L production reactor. Moreover, an earlier shift from a growth associated to a production stage associated gene expression pattern was identified for uHSD cultures compared to the reference. Overall, we showed that the described intensification strategy yielded in a higher volumetric productivity and is applicable for existing or already approved molecules in common, commercial fed-batch facilities. This work provides an in-depth molecular understanding of cellular processes that are detrimental during process intensification.

**Electronic supplementary material:**

The online version of this article (10.1007/s00449-020-02337-1) contains supplementary material, which is available to authorized users.

## Introduction

Monoclonal antibodies (mAbs) and other derived therapeutic proteins are currently one of the most important product classes in the pharmaceutical industry and will likely remain relevant for many years to come [1, 2]. Typical process formats comprise mammalian expression systems such as Chinese Hamster Ovary (CHO) cells cultivated in fed-batch processes with final production scales of several cubic meters [3]. Increasing market demands for monoclonal antibody therapeutics and the pressure for cost-reduction in their production [4, 5] have led to new manufacturing approaches and improved facility utilization strategies e.g., continuous production [6, 7]. For implementation of upcoming process intensification technologies, pharmaceutical companies can aim for scale out by building additional manufacturing capacities or for intensification in existing pharmaceutical production plants. Both strategies require substantial investments and the changes have to be balanced against cost and downtime during installation and subsequent re-qualification of equipment. In the case of fully continuous production processes, an implementation in existing fed-batch facilities is often impossible due to open technical and regulatory questions such as higher complexity and increased media amounts [7–9]. A smart step for the development and maintenance of marketable antibody portfolios is to search for intensification strategies, which debottleneck production chains using available capacities whenever possible and which are implementable in existing facilities, such as *N* − 1 perfusion [10].

The concept of pre-stage (*N* − 1) perfusion shifts biomass production away from capital-intensive large-scale reactors into the smaller pre-stage systems, as described by Kloth et al. [10]. Cell retention combined with media exchange facilitates robust exponential cell proliferation without any loss of biomass and can be achieved by a number of technologies, which differ in robustness and scalability [11]. Perfusion processes have the potential to reach over 100 × 10^6^ cells/mL [12], so that the required biomass generation of several sequential batch pre-stages can be combined into one perfusion stage. Consequently, the number and size of seed bioreactors could be reduced shortening overall process timelines. Reaching a higher biomass in perfusion becomes feasible due to continuous removal of growth-inhibiting factors that usually accumulate during growth [9], in combination with optimized feeding through adjusted cell-specific perfusion rates (CSPR) [13]. Furthermore, the *N* − 1 perfusion concept allows much higher seeding of production bioreactors aiming for shorter process runtimes or higher final product concentrations. Examples for pre-stage perfusion of CHO antibody processes have been published in recent studies [14–17].

Furthermore, these studies have confirmed the application of increased biomass from pre-stage cultivation for high seeding cell density in production scale to improve monoclonal antibody yields and decrease process times in intensified processes.

Mainly, the increased space–time–yield is achieved through faster progression from growth into stationary production phase in the final bioreactor scale with optimized medium. This concept, often referred to as concentrated fed-batch or ultra-high seeding density process (uHSD), has shown particular potential in combination with media and feeding development strategies, e.g., as reported by Yang et al. [18, 19]. Hybrid strategies with perfusion and cell-dependent feeding strategies as shown by Hiller et al. [20] further developed the concept towards fully continuous production, while still being applicable to existing production facilities with moderate refitting efforts. The target of media and feeding optimizations is to compensate any limitations of platform media that result from higher cell density.

The present work combines pre-stage perfusion with tangential flow-filtration (TFF) based cell retention and ultra-high seeding cell density in production culture with the target to maintain product quality, increase volumetric productivity and thus overall intensify a reference CHO fed-batch process for monoclonal antibody production.

On that path, we obtained deeper process insights by mechanistic modelling of perfusion cultures and transcriptome profiling using next-generation sequencing (NGS) to allow for a systematic analysis and adaptation of the process intensification strategy.

## Material and methods

### Cell lines, culture media

Cell culture experiments were performed using a CHO-DG44 derived cell line producing a fully humanized, monoclonal IgG1 antibody with a molecular weight of approximately 150 kDa. Growth, production and feed media derived from the proprietary BI-HEX^®^ platform were used for this work. A modified standard medium was established for perfusion cell cultivation and the subsequent final stage (*N* stage) high seeding density set-ups. All media components and reagents were acquired in cell culture or pharmaceutical grade.

### Seed cultures

For seed cultivation, cells were thawed and passaged in shake flasks (Corning, USA) every 3 days with incubator settings of 120 rpm (50 mm orbital throw), 36.5 °C and 5% CO_2_. For lab-scale perfusion (4 L) four passages were performed. For seed cultivation of pilot-scale perfusion (20 L) cells were passaged eight times and expanded in shake flasks, wave-mixed bioreactors (Sartorius AG, Germany) and glass-stirred tank bioreactors (STBRs) in batch-mode (Diemar Glasgeräte Wertheim, Germany).

### Perfusion seed cultures

Cells from the seed cultures were used to inoculate *N* − 1 perfusion cultures. Lab-scale perfusion was conducted in a 4 L glass STBR (Schmizo AG, Germany) with a working volume of 3.1 L and coupled to an external hollow fiber filter for cell separation in TFF mode. For scale-up to pilot-scale, *N* − 1 perfusion was performed in a 20 L STBR (Mavag AG, Germany) with 16 L working volume using the KML 100 System (Spectrum Laboratories, Inc., USA). Regardless of scale, cells were pumped through the external filter bypass by the Levitronix centrifugal pump PuraLev 200 (Levitronix GmbH, Switzerland) with magnetic bearings, which was set to a constant perfusion rate of 0.4 L/min to obtain a residence time < 1 min for cells in the bypass. The perfusion rates were automatically controlled via a scale-based feedback control loop. Pore sizes of 0.2 or 0.65 µm were used to retain the cells in the bioreactor system and to wash out byproducts and product. Perfusion culture duration was 5–6 days (until sufficient amount of cells were reached to seed uHSD cultures) including an additional initial batch phase of one day. Temperature was controlled at 37 °C and pH between 7.2 and 6.8 applying CO_2_ sparging and 1 M sodium carbonate solution. At process end cells from *N* − 1 perfusion were used to inoculate reference and concentrated fed-batch production cultures.

### Fed-batch production cultures

Lab-scale fed-batch production cultures were performed in 2 L glass bioreactors (Boehringer Ingelheim Pharma GmbH & Co. KG, Germany) while for scale-up to pilot-scale an 80 L STBR (Applikon Instruments, the Netherlands) was used. Concentrated fed-batch processes inoculated with an ultra-high seeding density (uHSD) of 10 × 10^6^ cells/mL were compared to controls, i.e. the reference processes inoculated with 0.7 × 10^6^ cells/mL. After an initial batch phase, nutrient feed was added to control cultures, whereas concentrated fed-batch cultures were fed immediately after inoculation until process end. Regardless of scale or seeding cell density, culture duration was 11 days, temperature, DO and pH were controlled at platform conditions. If necessary, additional glucose boli were added to maintain optimal glucose concentration in the culture. For one 2 L lab-scale uHSD run a bolus shot of sodium lactate, was tested on day six; in pilot-scale (80 L) a sodium lactate stock solution was used to maintain lactate concentration high enough to favor lactate consumption.

### Analytical methods

Bioreactor cultures were sampled daily. Cell counts and viabilities were determined using the automated CEDEX Analyzer MS20 C (Roche Diagnostics, Germany). Metabolic parameters including not only glucose, lactate, ammonia, glutamine and glutamate but also the immunoglobulin G titer concentration were quantified via the Konelab™ Prime 60i system (Thermo Fisher Scientific Inc., USA). pH, pO_2_ and pCO_2_ were measured offline with Rapidlab™ 348 blood gas analyzer (Siemens Health Care GmbH, Germany). Osmolality was monitored with OSMOMAT^®^ auto (Gonotec GmbH, Germany). For product quality analysis, harvested cell free culture fluid was processed through a 1-mL scale protein A capture step. Subsequently, ultra-performance size exclusion chromatography (UPSEC) via ACQUITY UPLC H-Class Bio (Waters Corporation, USA), imaged capillary electrophoresis (iCE) using iCE280 (ProteinSimple, USA), weak cation-exchange chromatography, capillary gel electrophoresis (cGE) and oligomap analysis measured via Caliper GXII (Perkin Elmer Inc., USA) were performed.

### Transcriptome profiling

For each sampling point 5 × 10^6^ cells were collected and total RNA was extracted using Trizol reagent. For mRNA purification and fragmentation as well as cDNA library generation, the TrueSeq RNA Sample Prep Kits v2-Set B (Illumina Inc., USA) was applied. The TruSeq SR Cluster Kit v3-cBot-HS (Illumina Inc, USA) was used for cluster amplification of the DNA library samples and single-read sequencing was carried out with the Illumina HiSeq3000-instrument and the TruSeq SBS Kit HS-v3 (FC-401-3002, Illumina Inc, USA). For NGS analysis Genedata Selector^TM^ and Profiler^TM^ (Genedata AG, Switzerland) were used for read mapping on an internally available, annotated CHO DG44 genome as well as for data processing. Genes were counted as significantly deregulated if the expression fold change was equal to or higher than two and if the Benjamini–Hochberg (BH) *p* value for multiple testing was lower than 0.05 [21]. Effect and network analysis were performed with QIAGEN Ingenuity Pathway Analysis (IPA) summer release 2016 (IPA^®^, QIAGEN, USA, https://www.ingenuity.com/).

### Modelling

For in-depth evaluation of perfusion process data, a modified mechanistic model based on a publication by Frahm [22] was used which was further extended by mass balances for a perfusion process. The parameter and variables lists for the modified mechanistic model are given in the Supplementary Tables 1 and 2. Additionally, the mass balances and kinetics are described in the supplementary material. Ordinary differential equation (ODE) terms were solved with Berkeley Madonna software V8.09 [23].

## Results and discussion

### Pre-stage perfusion in 4 L lab-scale and 20 L scale-up

**Fig. 1:**
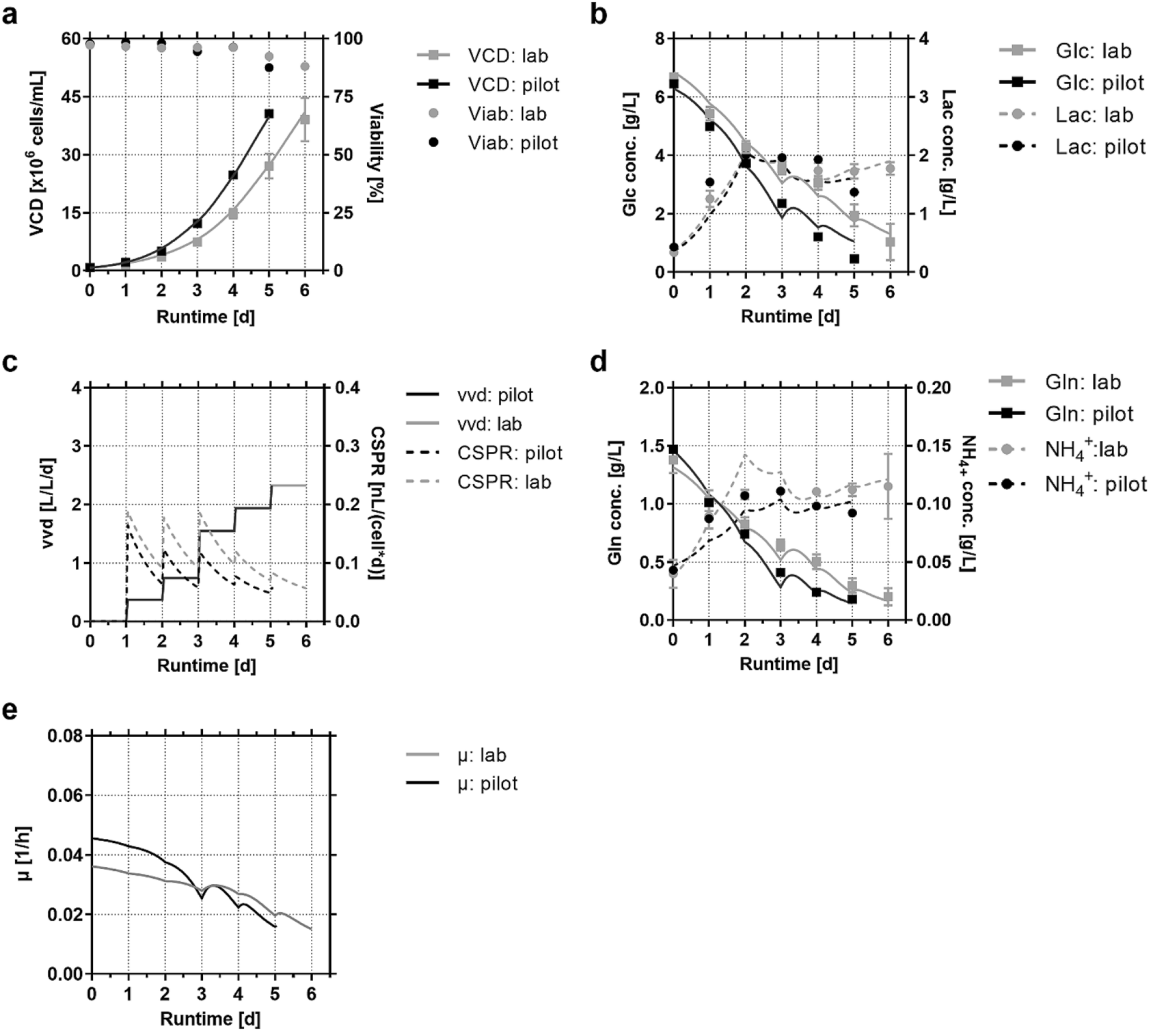
* N* − 1 pre-stage perfusion cultures. For 4 L lab-scale runs (*n* = 5 by day 4, afterwards *n* = 4 due to technical issue) and 20 L pilot-scale (*n* = 1) the following parameters were measured (symbols) and plotted versus the process model (lines). **a** Viable cell density (VCD) and viability (Viab), **b** the metabolites glucose (Glc), lactate (Lac) in supernatant, **c** the cell-specific perfusion rate (CSPR) and exchanged volumes per day (vvd), **d** glutamine (Gln) and ammonium (NH_4+_) in supernatant. In **e** the growth rate (*µ*) is shown

Perfusion in the *N* − 1 stage was first established in a 4 L laboratory reactor with 3.1 L working volume (0.1 L account for the flow path fill). The setup used a modified growth medium with volumetric exchange rates increasing daily from 0.4 to 2.3 volumes of medium per reactor volume per day (vvd). This corresponds to approximately seven exchanged bioreactor volumes at process end, which allows direct transfer to typical commercial facilities offering a 1:7.5 ratio of pre-stage bioreactor volume to media storage tanks. Figure 1 shows the growth and metabolic data of the CHO DG44 cell line fitting well to the perfusion process model. The model considered mandatory constraints for large-scale implementation and was used to describe the time course for important process parameters like growth and main substrate consumption rates in order to evaluate effects of the process format on the perfusion culture. For *N* − 1 perfusion, peak cell densities up to 45 × 10^6^ cells/mL were achieved within six process days. Growth and substrate concentration showed sufficient supply conditions throughout the perfusion process, which is also confirmed by amino acid measurement from supernatant (no limitations detected, data not shown). The targeted mean cell-specific perfusion rate (CSPR) for the perfusion process was 0.12 nL/cell/day. In order to facilitate implementation in large scale, not an exponential but a simplified, daily-adjusted step profile was applied, resulting in a range of 0.04 and 0.19 nL/cell/day also reported in literature [13]. Although the growth rate and metabolite profiles decreased over process time indicating a non-perfect steady state, the transferability of the generated biomass for inoculation of uHSD *N* stages was confirmed in several replicates.

For scale-up to pilot-scale, the same medium was used and volume-specific exchange rates were kept constant. Slightly higher growth rates were observed. Thus > 40 × 10^6^ cells/mL was already reached after five process days. As a result, lower average CSPRs were detected and glucose was limited on day five resulting in a slight drop in cell viability. This improved performance for pilot-scale can likely be attributed to flow path optimization in the pilot system: bottom-level drainage was implemented for the TFF bypass, whereas the lab-scale had immersion pipe drainage. Both, the lab- and pilot-scale systems relied on magnetically levitated centrifugal pumps, which were designed for low shear stress. Therefore, the increased geodetic height of the top drainage pipe trigged higher centrifugal pump settings and thus higher shear forces in the lab-scale system. Shear rates in lab-scale were kept below 1100 1/s which is already lower than reported in literature by Lin et al. [24]. However, for pilot-scale the shear rate was kept even lower at approximately 400 1/s, which could contribute to the improved performance.

In summary, the pre-stage perfusion effectively shifted biomass production to the pre-stage and was able to generate sufficient amounts of viable cells for inoculation with ultra-high seeding cell densities (uHSD) of 10 × 10^6^ cells/mL in the *N* stage production processes. As outlined by Blaschczok et al. [25], further scale-up for centrifugal pumps is possible and connection via bottom port is clearly favorable to minimize shear rates.

### Concentrated fed-batch production cultures

First iterations of the uHSD lab-scale process (uHSD v0.1 runs) were performed to identify media components that improve transferability and metabolic properties from *N* − 1 to *N* stage in comparison to a reference process. Thus, the metabolites lactate and l-glutamine in the *N* stage production medium were adjusted to mimic the concentrations on the last process day in the *N* − 1 stage. The purpose was not only to improve transferability but also to avoid extensive media development in this study for various reasons like, e.g. regulatory acceptance, need of new base powder sourcing or need to characterize new media stabilities. While no effect on the cell culture performance was observed (Supplementary Fig. 1), the transcriptome revealed a reduced genetic deregulation during culture transfer for the uHSD run including media adaptations compared to the v0.1 variant using the platform medium. This result is probably explainable by a less stressful environment. The details of the transcriptomic analysis will be part of a later section. However, it resulted in the adapted, non-platform production medium for the uHSD setup, which formed the basis for the following process development approach.

**Fig. 2:**
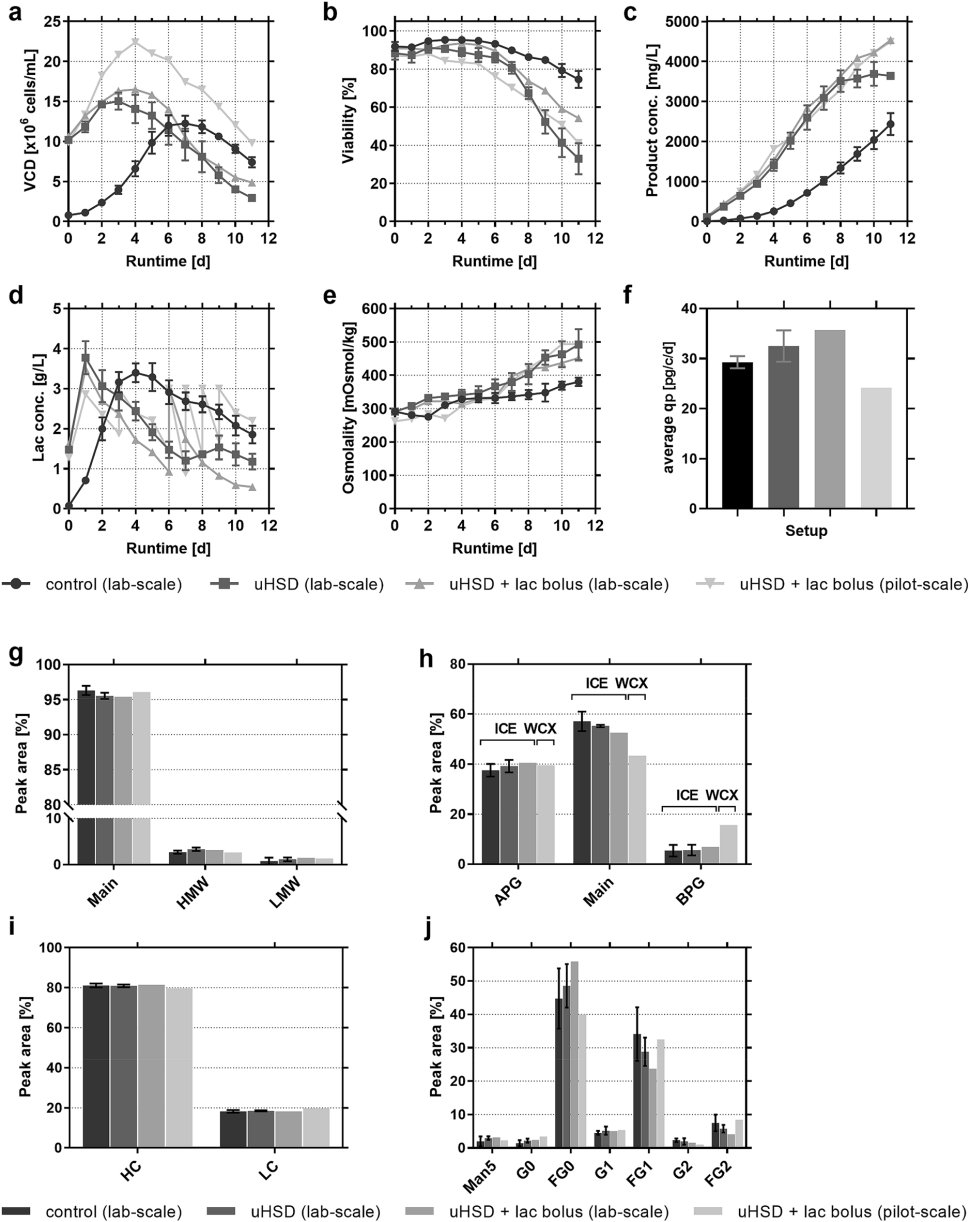
Fed-batch production cultures. Top panel: *N* stage cell culture data including **a** viable cell density (VCD), **b** viability, **c** product concentration, **d** metabolite lactate, **e** osmolality and **f** average cell specific productivity *q*_p_. Bottom panel: product quality data for same cultivations, **g** integrity via UP-SEC, **h** charge patterns via ICE280 in lab-scale, WCX in pilot-scale, **i** reduced capillary gel electrophoresis, **j** glycosylation via oligomap analysis. Control (lab-scale): 2 L reference (*n* = 5); uHSD (lab-scale): 2 L concentrated fed-batch (*n* = 3); uHSD + lac bolus (lab-scale): 2 L concentrated fed-batch with lactate bolus on day 6 (*n* = 1); uHSD + lac bolus (pilot-scale): concentrated fed-batch in 80 L pilot-scale with lactate boli according addition criteria (*n* = 1). Error bars represent one standard deviation from mean, when *n* ≥ 3

Figure 2 shows the direct comparison of reference fed-batch controls and concentrated fed-batch processes using uHSDs in the 2 L lab-scale system as well as the scale-up approach to 80 L pilot. Harvest and product quality analytics were performed on the last process day. The results clearly show that the mAb product titer could be increased by 1.5-fold in an equivalent runtime using the uHSD concept. Notably, implemented lactate boli further improved the mAb product titer by 1.9-fold compared to reference process and demonstrated elevated final viability compared to the basic uHSD approach. According to the uHSD principle, titers were increased via an improved space–time-yield not only through faster progression from growth into stationary production phase but also via enhanced average cell-specific productivities (average qp, Fig. 2f). For the basic uHSD runs, a plus of 10% and for the fully developed version including a lactate bolus, a plus of 18% in average qp was achieved compared to the control. Lactate feeding to a mammalian culture process itself has been already reported to improve specific productivities [26, 27]. This effect might often be attributed to a hyperosmotic effect [28, 29]. In our study, uHSD runs generally showed a slightly higher osmolality in the later culture phase than the control runs with low seeding, which is mainly attributed to a higher feed rate. In contrast, additional lactate bolus additions did not further increase osmolality because of its rapid consumption by the cells (Fig. 2e). Interestingly, Becker et al. [30] reported an increase of 50% in cell-specific productivities due to glucose limitation, which was coupled to lower lactate formation. Similarly, we observed a low, but not limited, glucose concentration of approximately 1 g/L on culture day 1 (Supplementary Fig. 2), followed by a metabolic shift towards lactate consumption. Feeding of lactate actually increased lactate consumption rates leading to a higher final mAb titer. Overall, we assessed the lactate consumption phase as an advantage for cell culture performance in the concentrated fed-batch processes. These results also fit to the study of Luo et al. [31], who demonstrated that a metabolic shift from lactate production to lactate consumption leads to an improved process performance with respect to productivity and cell growth.

Scale-up to pilot-scale was successful achieving a comparable final mAb titer, but also observing an increased peak VCD, which entailed a reduced cell specific productivity. The scale-dependent offset could probably be a specific scale effect of this particular cell line in the given 2 L small-scale system. Due to the higher cell density, more lactate bolus additions were necessary compared to the run performed in a glass bioreactor.

Regarding the product quality attributes, mAb purity and integrity remained unaffected when comparing the control fed-batch to the uHSD processes (Fig. 2 g–j). The N-linked glycan structures were typical for the applied cell line with predominant A2FG0 glycan forms. A slight trend hints towards a negative correlation between product galactosylation and average qp explainable by an extended time span for the glycan structure to mature at lower production rates. Overall, the small differences are marginal and the results are considered to be comparable. Minor differences in charge variant distribution for the pilot-scale may be an analytical artifact, as different methods were applied for lab- (ICE) and pilot-scale runs (WCX). The observed robustness in the product quality attributes is in accordance with the observations made by Yang et al. [15], who reported that a seeding cell density dependence of product quality attributes can be present or absent depending on the respective CHO production clones applied. Nevertheless, specific setup differences may certainly also play a role (e.g., ATF instead of TFF, unknown bypass residence time, CSPR).

The overall potential for the uHSD version with lactate feeding is considered highly promising as the 1.9-fold titer could be reached in combination with a comparable product quality, without changing the cell line and in a process setup that fits to existing manufacturing facilities. These are key parameters allowing the application for new biological entities (NBEs), which have already reached clinical trials or have received market approval using a regular fed-batch process. With regard to the intended use, the developed strategy is superior to other known intensification approaches such as fully continuous production, which would have the potential to deliver a much higher volumetric productivity [32]. However, an implementation in existing fed-batch facilities is often impossible or at least much more challenging due to open technical or regulatory questions and due to comparability topics for existing projects.

### Capacity utilization

The present results confirm that pre-stage perfusion combined with concentrated fed-batch production has the potential to increase capacity utilization due to faster accumulation of biomass in the *N* − 1 stage and higher cell-specific productivity in the final bioreactor stage (*N* stage). Different operation strategies differentiate based on parameters such as facility structure, titer improvement and process length. First, runtime of the production bioreactor could be reduced by 45% to 6 days achieving a comparable titer as the reference fed-batch process in 11 days. Yang et al. [15] presented an empirical equation estimating the maximum number of manufacturing batches per year. It takes into account the process length of *N* − 1 seed cultures, turnaround times of the production bioreactors, process length of the production run, overall campaign length and the ratio of available production to seed vessels. Considering a widespread commercial facility design with a total of six large-scale production bioreactors and four seed trains [15], the number of batches per year can theoretically be increased by 14%. However, two further major aspects need to be considered: First, the turnaround time of the seed bioreactor has to be taken into account. Second, an additional tank is needed for perfusion-media storage, which takes up the equivalent of another large-scale production bioreactor in the facility. Moreover, the number of batches per year is rather limited due to bottlenecks in harvest and downstream procedure than by production bioreactor duration of ≤ 11 days.

Thus, another strategy is to increase the single-batch output by doubling the product concentration in an equivalent runtime as standard, low-seeded fed-batch cultures. In Table 1 the final product concentration of the low-seeded reference fed-batch is compared to the best-performing uHSD run, which included a lactate bolus. To produce the same amount of product over a year, the number of batches could be reduced by 46% from 147 standard batches to 79 intensified batches per year as batch productivity is improved from 2.1 to 3.9 kg/day.

**Table 1:** Effect of ultra-high seeding concept on capacity utilization

	Reference (control)	Intensified (uHSD + lac bolus)						
Runtime *N* stage (day)	11	5	6	7	8	9	10	11
Titer (g/L)	2.4	2.2	2.8	3.2	3.6	4.1	4.2	4.5
Batch productivity (kg/day)	2.1	3.3	3.8	3.8	4.0	4.1	3.9	3.9
Mass per batch (kg)	29.2	26.0	34.0	38.3	43.6	49.0	50.7	54.4
Increase of mass per batch (%)	n.a	− 11%	16%	31%	49%	68%	73%	86%
Batches per year (–)	147	165	126	112	99	88	85	79
Reduction of batches per year (%)	n.a	− 12%	14%	24%	33%	40%	42%	46%
Mass per year (kg)	4296	4296	4296	4296	4296	4296	4296	4296

The final product titer of the low-seeded reference fed-batch (control) was compared to the best-performing uHSD run, which included a lactate bolus. Numbers of manufacturing batches per year were calculated to evaluate the maximum degree of batch reduction for uHSD concept. For standard fed-batch processes 147 batches per year and for calculating the batch productivity a bioreactor turnaround time of 3 days were assumed

Considering all of the above aspects, our results indicate that for this particular layout the superior strategy for increasing the plant capacity is a reduction of manufacturing batches per year: Hence, almost half of the batches could be saved while maintaining the same product output.

For further improvement of the batch capacity, enhanced seeding cell densities for the concentrated fed-batches are conceivable like, e.g., 20 × 10^6^ cells/mL. However, for such an approach several constraints have to be considered: the limited runtime of the *N* − 1 perfusion to fit into existing facilities, maximum media exchange volumes during perfusion or restricted split-ratios for inoculation of the *N* Stage. In the described setup, 10 × 10^6^ cells/mL represents the maximum SCD including a safety range for *N* − 1 performance. Nevertheless, with further optimized *N* − 1 perfusion processes, even higher SCDs might be applicable for the concentrated fed-batches.

### Transcriptome profiling using next-generation sequencing (NGS)

Transcriptome profiling was performed for cells cultivated in *N* − 1 pre-stage perfusion and the *N* stage fed-batch processes with basic platform and adapted media formula (Table 2, cell culture data, see Supplementary Fig. 1a–e). Quantitative changes in gene expression levels were investigated via RNA deep sequencing (RNA Seq) with focus on the following aspects.

1. Transcriptomic analysis of the perfusion process, i.e., change of transcript levels during the *N* − 1 perfusion process.
2. Transcriptomic analysis during the cultivation in different *N* stage media formulations, i.e., adaptation of lactate and L-glutamine concentrations analogous to *N* − 1 on the last process day (uHSD) compared to the initial test with platform media formulation (uHSD v0.1).
3. Transcriptomic analysis during the cultivation in different *N* stage process setups, i.e., uHSD concept compared to reference fed-batch process.

**Table 2:** Overview of NGS samples with process setups and sampling points

Setup (lab-scale)	Stage	Media	Sampling point for RNA Seq (phase)
Perfusion	*N* − 1	Perfusion	Day 0 (L), day 3 (G), day 6 (G)
Control	*N*	Platform (fed-batch)	Day 0 (L), day 1 (G), day 4 (G), day 11 (D)
uHSD v0.1 (initial small scale test with uHSD)	*N*	Platform (fed-batch)	Day 0 (L), day 1 (G), day 4 (A), day 11 (D)
uHSD (basic small scale uHSD process)	*N*	Adjusted	Day 0 (L), day 1 (G), day 4 (A), day 11 (D)

Three sampling points (day 0, day 3 and day 6) for *N* − 1 pre-stage perfusion were chosen, while in fed-batch production processes four sampling points (day 0, day 1, day 4, day 11) were selected. Thus, all process phases [lag (L), growth (G), growth arrest (A) and decline phase (D)] are covered

**Fig. 3:**
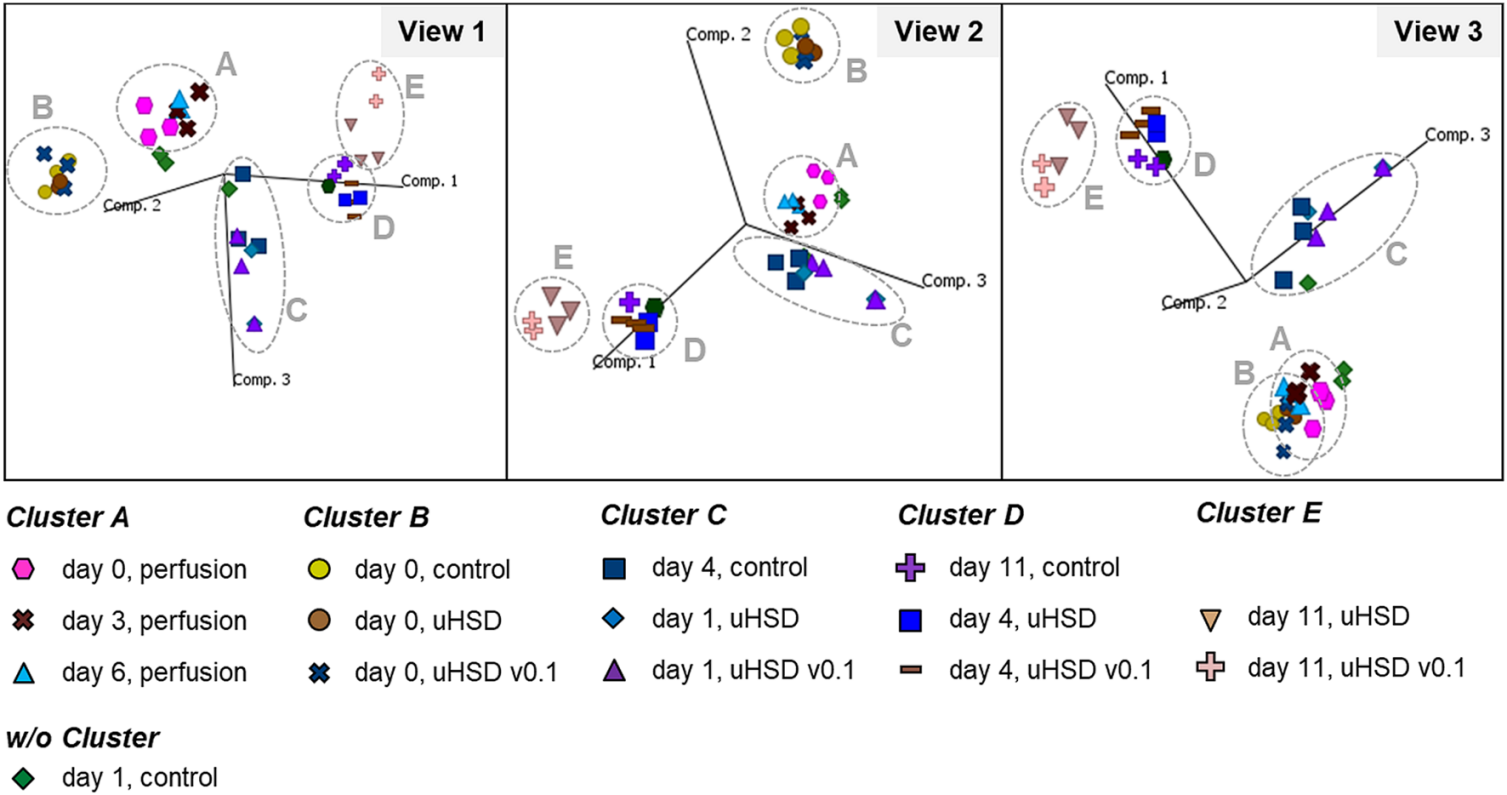
Principal component analysis (PCA) for gene expression data from next-generation sequencing. Datasets are derived from *N* − 1 perfusion (*n* = 3 technical replicates), control fed-batch process (*n* = 2 biological replicates and *n* = 1 technical replicate), initial test with uHSD (uHSD v0.1 with platform medium, *n* = 2 biological replicates and *n* = 1 technical replicate) and final uHSD process (uHSD with modified medium, with *n* = 2 technical replicates)

A principal component analysis (PCA) was performed from all acquired transcriptome datasets in order to allow for an overall comparison of the different gene expression profiles. Principal components are linear combinations of possible variables among mRNA abundance patterns, which can be used to explain the variance in gene expression data. Figure 3 presents the results for the first, second and third principal component. Clustering showed that perfusion samples throughout the pre-stage had similar gene expression patterns indicated by the formation of a cluster (Cluster A) demonstrating mostly steady-state transcription levels within the *N* − 1 perfusion stage. Another cluster was formed for the transfer to the uHSD *N* stage production bioreactor, where there is no immediate impact of seeding density (Cluster B, day 0 samples)—thus, gene expression patterns are likely to be similar. What follows is a sequential progression of gene expression patterns that depends on time and seeding density: uHSD v0.1 and uHSD cultures moved rapidly through Clusters C–E with each sampling day, whereas the control process samples clustered in the same regions but had a delayed response. This suggests that culture phases are expedited in the uHSD approach, which is in good accordance with cell growth and mAb product titer data (Supplementary Fig. 1c–e).

**Fig. 4:**
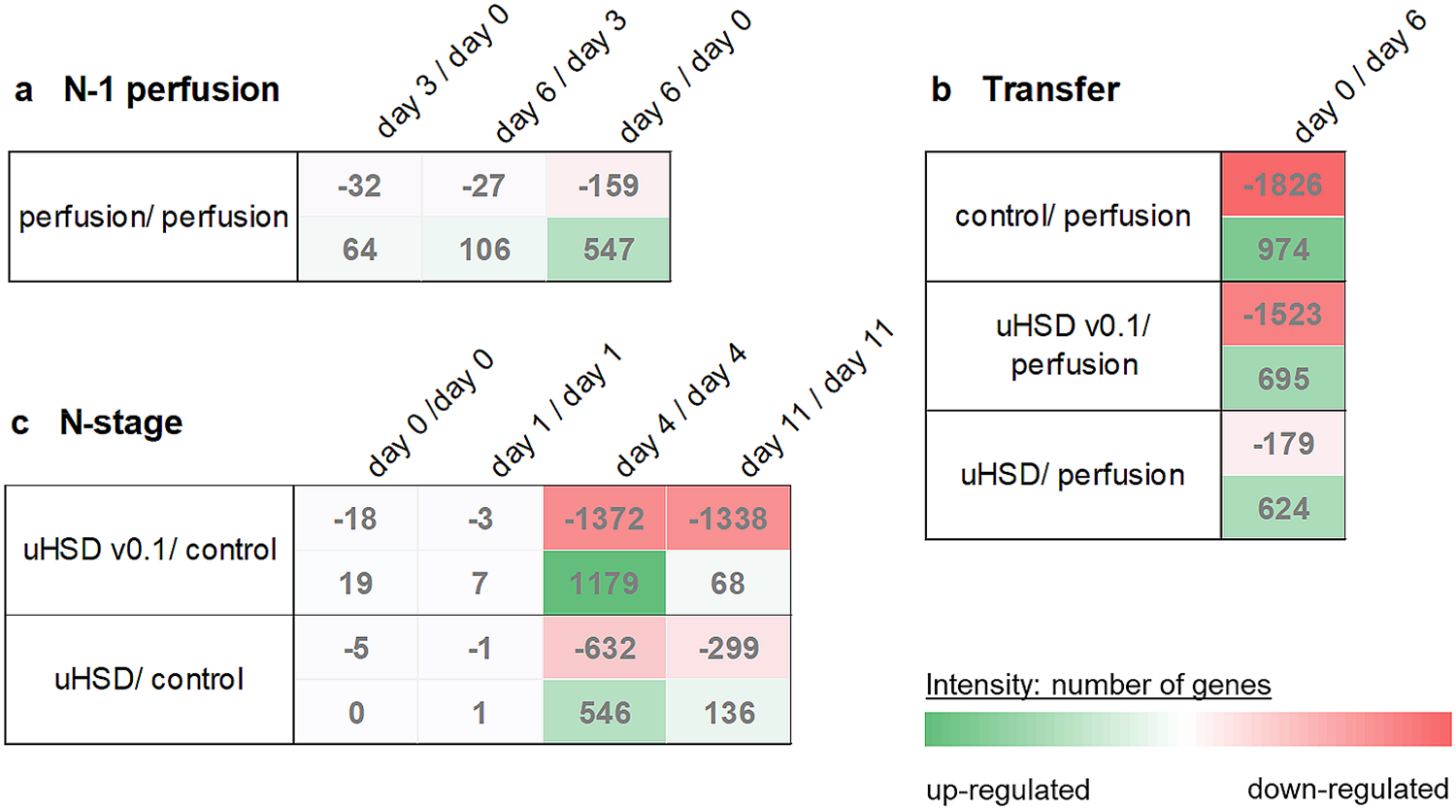
Overview of total count of differentially expressed genes during **a***N* − 1 perfusion, **b** transfer from *N* − 1 perfusion to *N* stage and **c***N* stage fed-batch. The datasets from *N* − 1 perfusion, reference fed-batch process (control), initial version of ultra-high seeding cell density process (uHSD v0.1) and ultra-high seeding cell density process (uHSD) with modified medium to minimize transfer changes are compared with respect to specified process days

The PCA analysis was further amended by comparing the number of differentially expressed genes between individual conditions (Fig. 4). There were low numbers of differentially expressed genes among the samples from the *N* − 1 perfusion culture, which might be evident for largely stable perfusion culture conditions. However, because perfusion cultures are not perfectly in a steady-state it was not surprising that at least some genes were found deregulated over the process time. This became especially visible at the last day of the *N* − 1 perfusion culture and was well in agreement with changing cell specific rate terms, for example the gradually decreasing growth rate *µ* (compare Fig. 1e).

**Fig. 5:**
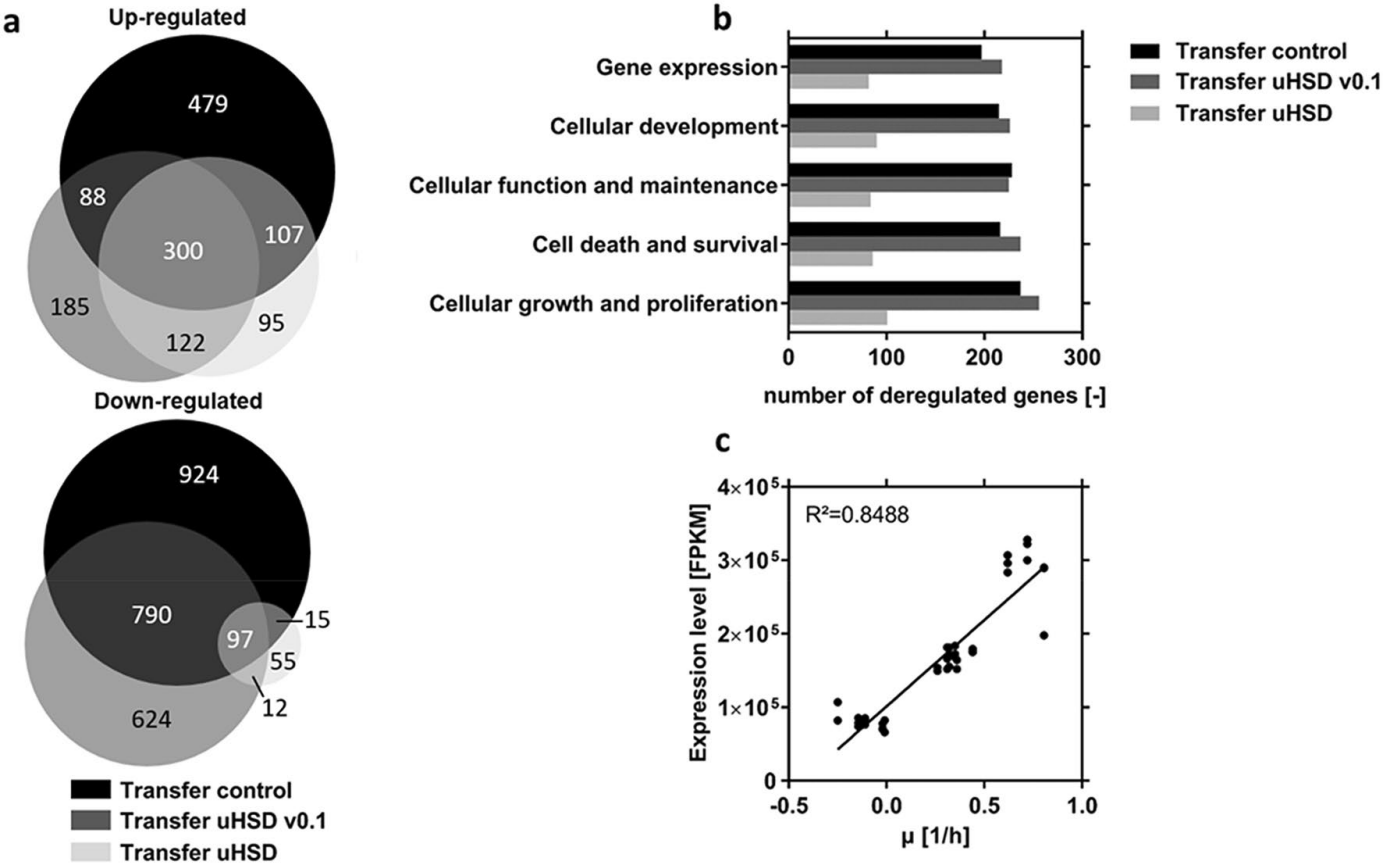
Analysis of deregulation during transfer. **a** VENN diagram for up- and down-regulated genes during transfer (day 0 of respective *N* stage versus day 6 of *N* − 1 perfusion) illustrating conclusive and exclusive deregulated genes for the investigated setups. **b** Ingenuity pathway analysis revealing the top five biological functions affected by transfer. **c** Expression levels of the transcription factor E2F1 over growth rate µ for all production stage cultures (*N* Stage) and the *N* − 1 perfusion culture (d0, d3). Curve for linear regression is shown (for calculation each expression replicate value was considered as an individual point)

The highest number of deregulated genes was detected during the transfer from the *N* − 1 perfusion to the *N* stage fed-batch production bioreactors, both for control and uHSD conditions (Fig. 4b). Moreover, we could demonstrate that media optimization can decrease the amount of differentially expressed genes during the transfer from *N* − 1 to *N* stage by 64%, when the final uHSD process is compared to the uHSD v0.1 setup. This is a strong indication that the implemented changes in the media composition reduced transfer stress for the cells and thus kept changes in culture conditions at a minimum. The VENN diagrams shown in Fig. 5a present the total number of differentially expressed genes (up- and down-regulation) during the transfer from *N* − 1 to the investigated *N* stage bioreactor setups. The total number of differentially expressed genes was slightly below 4000. However, the high number of commonly deregulated genes, as shown by the overlapping area, revealed common changes most probably attributed to the transfer procedure in general rather than to an impact of the seeding cell density. Functional clustering analysis of the conclusive deregulated genes (overlaps) using Ingenuity Pathway Analyzer (IPA, Fig. 5b) indicated changes in central signaling networks boosting viability, higher proteome turnover and gene expression which is entailed by all transfers. This suggests that all cultures were reactivated during transfer from *N* − 1 to *N* stage and further emphasizes that conditions at the last day of the perfusion preculture no longer supported perfect exponential cell growth. Hence, a thorough fine-tuning of the transfer conditions and runtime of the perfusion culture are essential for a smooth scale up towards the *N* stage production bioreactor.

Over the entire culture duration of the *N* stage bioreactors a lower number of genes was found to be differentially expressed in the uHSD setup with media optimization, as compared to the initial uHSD version using the platform fed-batch media formulation (Fig. 4c). However, PCA did not indicate differences between different media formulations in uHSD setups. This could be due to the overall comparable cell culture process regardless of the media compositions. For the evaluation on potential effects of the seeding cell density, the datasets were compared on a culture day-by-day basis (Fig. 4c). Few genes were differentially expressed for inoculation samples (day 0, day 1). These results strongly suggest that there is no immediate change in gene expression patterns between control and uHSD culture conditions within the first two process days. Further, it is obvious that the rapid decrease in cell viability in advanced uHSD processes might have an impact on differential gene expression analysis due to mRNA degradation. Therefore, our focus was on the comparison of the fourth process day. IPA analysis of the differentially expressed genes on day 4 of the uHSD versus the control process indicated that uHSD conditions affected central cellular biological functions such as decelerated cell cycle progression or cell cycle arrest, increased DNA damage, decreased corrective activity against DNA damage, increased apoptosis and decrease in viability (Supplementary Figs. 3–5). This is in good accordance with the growth observations that the uHSD process has reached the late stationary phase already on day 4.

We then searched for central regulators of the observed cell phenotypes among these differentially expressed genes. Thereby, the transcription activator E2F1 was discovered as potential regulator of cell culture performance in uHSD cultures in dependence on seeding cell density. E2F1 was down-regulated in both uHSD setups compared to the reference process while an increased number of E2F1 binding motives were found in the upstream DNA regions of other down-regulated genes (Supplementary Table 3). The IPA regulator analysis also identified E2F1 as a potential regulator that might be responsible for the observed gene expression changes in the dataset. This analysis considered causal effects described in the literature, which are compiled in the Ingenuity Knowledge Base. The top ten of the most deregulated E2F1 target genes are listed in Table 3 including the respective biological pathways of their translated proteins. These potentially influenced pathways fitted the literature defining E2F1 as a transcriptional activator that is involved in the regulation of cell cycle, cell proliferation and apoptosis as well as it fitted the experimental data in this study. As shown in Fig. 5c, a correlation between E2F1 expression and the growth rate µ can be drawn in the dataset connecting cell culture performance to E2F1 as a potential regulator. The role of E2F1 has been confirmed previously by Majors et al. [33], who have demonstrated that overexpression of E2F1 can lead to improved viable cell density of CHO cells grown in batch cultures.

**Table 3:** Top ten of the most deregulated target genes of E2F1 described in the literature compiled in the ingenuity knowledge base

Gene symbol	Log2 FC uHSD v0.1/control	Log2 FC uHSD/control	Ingenuity knowledge base	Binding motif	Biological process
Aurkb	− 4.8	− 3.9	×	–	Cell cycle, cell division, mitosis
Ccna2	− 4.7	− 4.0	×	–	Cell cycle, cell division, mitosis
Ccnb2	− 4.4	− 3.6	×	×	Cell cycle, cell division, mitosis
Mcm10	− 4.2	− 3.2	×	–	DNA damage, DNA replication
Mybl2	− 4.2	− 3.5	×	–	Transcription, transcription regulation
Ect2	− 4.2	− 3.5	×	–	Cell cycle, cell division, differentiation, neurogenesis, protein transport, transport
Top2a	− 4.0	− 3.4	×	–	Chromatin organization
Rad54l	− 3.9	− 2.8	×	×	DNA damage, DNA repair
Birc5	− 3.8	− 2.5	×	–	Apoptosis, cell cycle, cell division, chromosome partition, mitosis, transcription, transcription regulation
Cdk1	− 3.6	− 3.0	×	–	Apoptosis, cell cycle cell division, mitosis

The Log2-fold change (FC) of the respective genes in both uHSD approaches compared to the low-seeded reference runs and the biological pathway of the translated proteins are listed

One of the above-mentioned down-regulated and putative target genes of E2F1 is Sirt-1, which is a histone deacetylase that is described to inhibit p53 in human [34]. Protein sequence homology of human Sirt-1 to the *Cricetulus criseus* (Chinese hamster) variant was found to be 90%. Our obtained data set also confirmed that 160 target genes of p53 were differentially expressed in a significant, conclusive manner that predict p53 to be activated (*p* value = 2.99E−45, Supplementary Table 4). Consequently, these results match the hypothesis of E2F1-dependent regulation of cell growth and viability in the *N* stage bioreactors. In addition, the genes of the E2F1 transcription regulators identified via upstream DNA binding sites (i.e., Elf5, Elk1 and SpiB) were not differentially expressed (Supplementary Table 5). These observations make E2F1 the most probable central trigger of transcription regulation, which may itself be regulated by other mechanisms (e.g., microRNAs or long non-coding RNAs).

## Conclusions

In conclusion, our study provides an in-depth analysis of the process performance, product quality and gene expression during cultivation of CHO production cells in *N* − 1 pre-stage perfusion as well as in the production bioreactor with and without ultra-high seeding density. A pre-stage perfusion strategy relying on TFF was successfully implemented for intensification of the seedtrain by shifting biomass production from *N* stage towards the pre-stage bioreactor, reaching 45 × 10^6^ cells/mL. This strategy enabled ultra-high seeding densities in the production stage, which increased overall volumetric productivity. In combination with process optimization strategies like a lactate bolus, mAb product titer was increased 1.9-fold for uHSD cultures in equivalent runtime without major impact on the product quality. Hence, this intensification strategy meets all mandatory requirements to be implemented for already existing projects (e.g., NBEs, which have reached clinical trials or received market approval already) due to comparable product quality, an unchanged cell line and its fit to existing manufacturing facilities. Furthermore, this approach enhances facility usage by facilitating the reduction of manufacturing batches per year by 46% via almost doubling the single-batch output. The successful scale-up to pilot-scale further demonstrated the potential of this strategy for industrial application. To the authors’ knowledge, this study constitutes the first published comprehensive process intensification through pre-stage perfusion and ultra-high seeding density, which also considers transcriptome assessment by NGS in combination with process performance evaluation. Using NGS, a stable gene expression pattern was identified for *N* − 1 pre-stage perfusion, while media adaptations reduced the number of deregulated genes during transfer of seed culture from *N* − 1 to *N* stage. Furthermore, an earlier shift from growth-associated to production stage-associated gene expression patterns was identified for uHSD cultures compared to the control fed-batch process. Transcriptome profiling data indicate that the accelerated culture phase progression and earlier dying phase in uHSD conditions might be attributed to a down-regulation of E2F1, which potentially acts as a key regulator of cell cycle progression, cell proliferation and apoptosis. In order to confirm these observations, it would be reasonable to stably overexpress E2F1 in CHO cells in order to functionally validate the regulatory role of E2F1 in uHSD production processes.

## Electronic supplementary material

Below is the link to the electronic supplementary material.

Supplementary file1 (DOCX 1063 kb)

Supplementary file2 (XLSX 10547 kb)

Supplementary file3 (XLSX 19570 kb)
