# Supplementary material for: Pre-stage perfusion and ultra-high seeding cell density in CHO fed-batch culture: a case study for process intensification guided by systems biotechnology
Source: Bioprocess Biosyst Eng. 2020 Apr 7;43(8):1431–43. doi: 10.1007/s00449-020-02337-1 (PMC7320070; doi:10.1007/s00449-020-02337-1)
Supplement: Supplementary file 1 — Supplementary file1 (DOCX 1063 kb) [file 449_2020_2337_MOESM1_ESM.docx]

**Electronic Supplementary Material**

**Pre-stage perfusion and ultra-high seeding cell density in CHO fed-batch culture: A case study for process intensification guided by systems biotechnology**

Bioprocess and Biosystems Engineering

Lisa Stepper^1^, Florian Alois Filser^1^, Dr. Simon Fischer^1^, Dr. Jochen Schaub^1^, Dr. Ingo Gorr^1^, Dr. Raphael Voges^1*^

^1^Bioprocess Development Biologicals, Boehringer Ingelheim Pharma GmbH & Co. KG, Biberach an der Riß, Germany

***Corresponding Author**

Dr. Raphael Voges: [raphael.voges@boehringer-ingelheim.com](mailto:raphael.voges@boehringer-ingelheim.com)

**Supplementary Process Data**


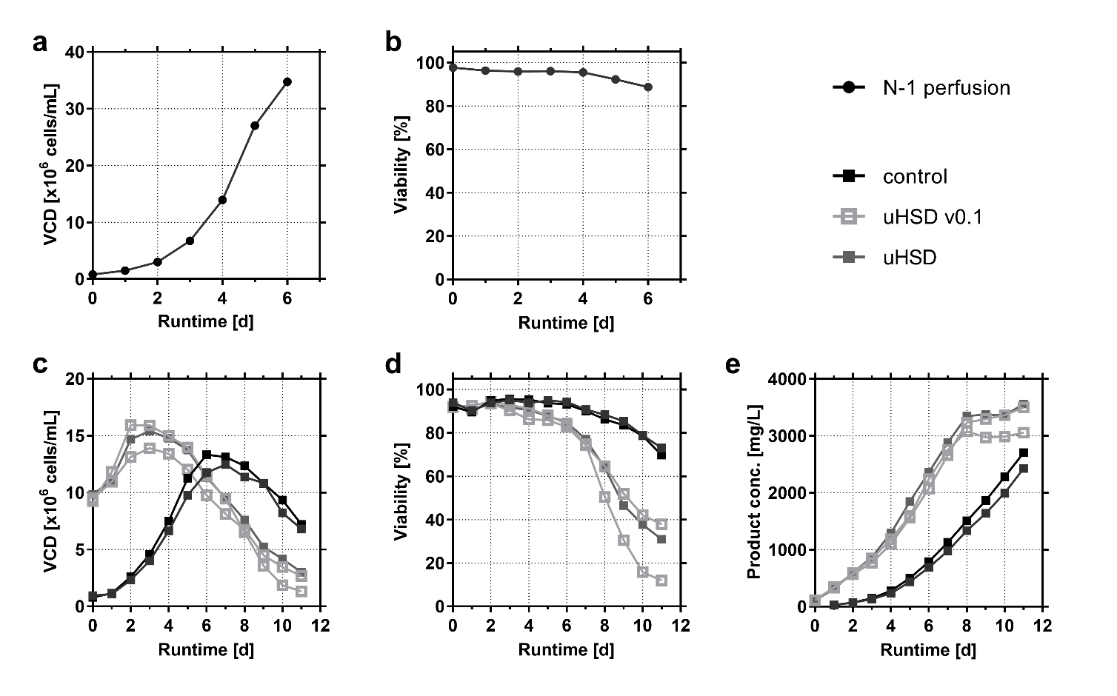


Supplementary Figure 1 First experimental iterations for media development and NGS analytics. Top panel: N-1 perfusion culture data including (a) viable cell density (VCD), (b) viability. Bottom panel: N-Stage culture data including (c) viable cell density (VCD), (d) viability and (e) product concentration for reference processes (control), initial test setups with 10 x10^6^ cells/mL (uHSD v0.1) and the final uHSD process (uHSD).


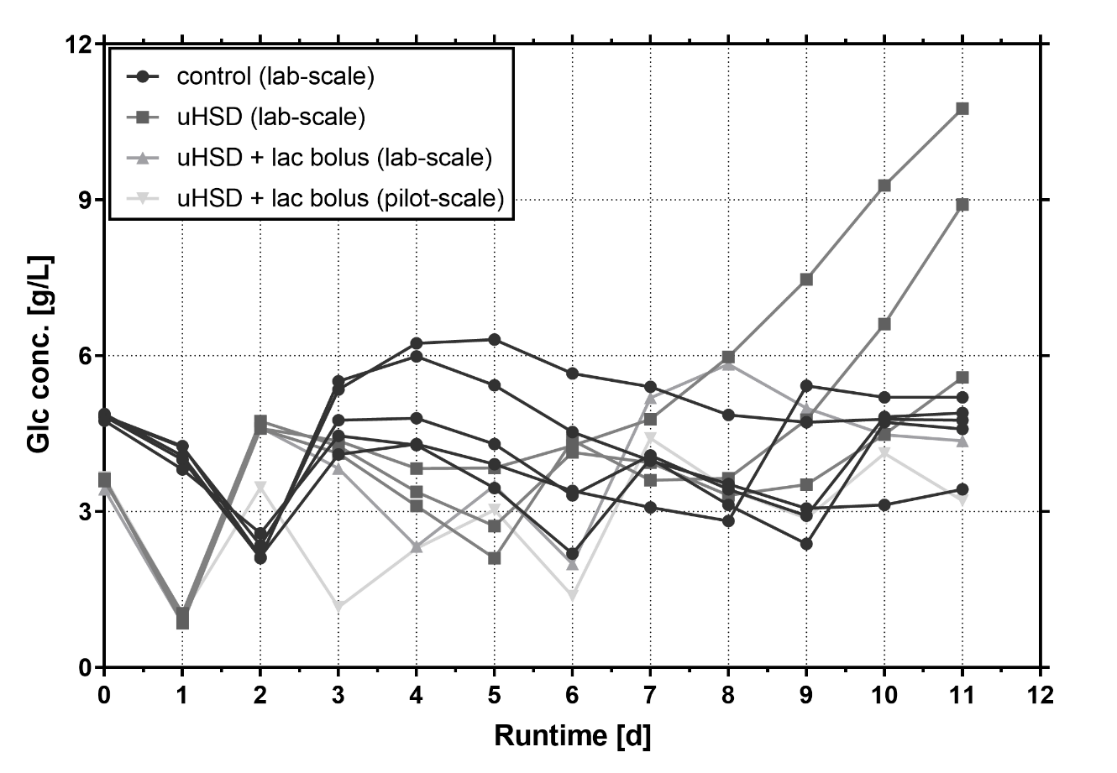


**Supplementary Figure 2 Glucose concentrations for fed-batch production cultures.** **Control (lab-scale): 2 L reference (n=5); uHSD (lab-scale): 2 L concentrated fed-batch (n=3); uHSD + lac bolus (lab-scale): 2 L concentrated fed-batch with lactate bolus on day six (n=1); uHSD + lac bolus (pilot-scale): concentrated fed-batch in 80 L pilot-scale with lactate boli according addition criteria (n=1). The data of the runs were not averaged as the glucose addition criterion allows an addition on different process days.**

**Modelling**

For in-depth evaluation of perfusion process data a modified mechanistic model based on a publication by Frahm B.^1^ was used which was further extended by mass balances for a perfusion process. The parameter and variables lists for the modified mechanistic model are given in the following section. Additionally, the mass balances and kinetics are described.

Supplementary Table 1 Parameter list for modified mechanistic model based on a publication by Frahm B^1^.

| **Parameter** | **Unit** | **Description** | **Value  20 L** | **Value  4 L** | **Origin** | **Sensitivity** |
| --- | --- | --- | --- | --- | --- | --- |
| $alpha$ | - | Parameter for higher glutamine consumption at low glucose concentration | 6 | 6 | [21] | Na |
| $c_{{NH4}_{t0}}$ | mmol/L | Initial ammonia concentration in the bioreactor | 2,60129 | 2,1 | Start conditions | Na |
| $c_{GLC_{t0}}$ | mmol/L | Initial glucose concentration in the bioreactor | 34,8975 | 38 | Start conditions | Na |
| $c_{GLC_{F}}$ | mmol/L | Glucose concentration in glucose feed | 1111,1 | 1111,1 | Start conditions | Na |
| $c_{GLC_{medium}}$ | mmol/L | Glucose concentration in media feed | 41,67 | 41,67 | Start conditions | Na |
| $c_{GLC_{border}}$ | mmol/L | Threshold for higher glutamine consumption at low glucose concentration | 0,5 | 0,5 | Start conditions | Na |
| $c_{GLN_{t0}}$ | mmol/L | Initial glutamine concentration in the bioreactor | 10 | 9 | Start conditions | Na |
| $c_{GLN_{F}}$ | mmol/L | Glutamine concentration in glucose feed | 100 | 100 | Start conditions | Na |
| $c_{GLN_{medium}}$ | mmol/L | Glutamine concentration in media feed | 12,33 | 12,33 | Start conditions | Na |
| $c_{{LAC}_{t0}}$ | mmol/L | Initial lactate concentration in the bioreactor | 4 | 4 | Start conditions | Na |
| $c_{{LIM}_{t0}}$ | mmol/L | Initial limiting substrate concentration in the bioreactor | 100 | 100 | Start conditions | Na |
| $c_{{LIM}_{medium}}$ | mmol/L | Concentration of Limiting substrate in media feed | 100 | 100 | Start conditions | Na |
| $c_{{mAb}_{t0}}$ | mg/L | Initial mAb concentration in the cell culture | 0 | 0 | Start conditions | Na |
| $k_{GLC}$ | mmol/L | Monod-constant for glucose uptake | 14,8 | 14,8 | Parameter fit | + |
| $k_{GLN}$ | mmol/L | Monod-constant for glutamine uptake | 1,7 | 1,7 | Parameter fit | ++ |
| $k_{LIM}$ | mmol/L | Monod-constant for limiting substrate uptake | 0,1 | 0,1 | [21] | Na |
| $k_{Lys}$ | mmol/L | Cell lysis constant | 0,1 | 0,1 | Parameter fit | - |
| $K_{p_{GLN}}$ | - | Correction factor for glutamine consumption at low glucose concentration | 1,2155 | 1,2155 | Parameter fit | +++ |
| ${K_{S}}_{GLC}$ | mmol/L | Monod-constant for glucose uptake | 1,5 | 1,5 | Parameter fit | ++ |
| ${K_{S}}_{GLN}$ | mmol/L | Monod-constant for glutamine uptake | 1,8 | 1,8 | Parameter fit | ++ |
| ${K_{S}}_{LIM}$ | mmol/L | Monod-constant for limiting substrate uptake | 0,01 | 0,01 | [21] | Na |
| ${mu}_{d_{min}}$ | 1/h | Minimum cell-specific death rate | 1,00E-04 | 0,001306 | Parameter fit | + |
| ${mu}_{max}$ | 1/h | Maximum cell-specific growth rate | 0,056071 | 0,045057 | Parameter fit | +++ |
| ${q_{GLC}}_{max}$ | mmol/(cell h) | Maximum cell-specific glucose uptake rate | 2,68E-10 | 2,78E-10 | Parameter fit | +++ |
| ${q_{GLN}}_{max}$ | mmol/(cell h) | Maximum cell-specific glutamine uptake rate | 5,23 E-11 | 5,23 E-11 | Parameter fit | ++ |
| ${k_{GLN}}_{deg}$ | 1/h | Chemical degradation constant of GLN | 0,0042 | 0,0042 | Media vendor | Na |
| ${q_{LIM}}_{max}$ | mmol/(cell h) | Maximum cell-specific uptake rate of limiting substrate | 1,36E-11 | 1,36E-11 | Parameter fit | - |
| ${q_{mAb}}_{max}$ | mg/(cell h) | Maximum cell-specific mAb production rate | 1,52E-09 | 1,52E-09 | Not evaluated | Na |
| $V_{t0}$ | L | Initial reactor volume | 16 | 3,1 | Start conditions | Na |
| $X_{t_{t0}}$ | Cells/L | Initial total cell concentration in the bioreactor | 8,50E+08 | 7,50E+08 | Start conditions | Na |
| $X_{v_{t0}}$ | Cells/L | Initial viable cell concentration in the bioreactor | 8,50E+08 | 7,50E+08 | Start conditions | Na |
| $Y_{NH4,GLN I}$ | mol/mol | Yield ammonia produced per glutamine in phase I | 0,7 | 1,4 | Parameter fit | + |
| $Y_{NH4,GLN II}$ | mol/mol | Yield ammonia produced per glutamine in phase II | 0,5 | 0,6 | Parameter fit | + |
| $Y_{LAC,GLC I}$ | mol/mol | Yield lactate produced per glucose in phase I | 1,2 | 1,4 | Parameter fit | + |
| $Y_{LAC,GLC II}$ | mol/mol | Yield lactate produced per glucose in phase II | 0,5 | 0,62 | Parameter fit | + |

^1^Frahm, B., *Seed train optimization for cell culture*, in *Animal Cell Biotechnology*. 2014, Springer. p. 355- 367

Supplementary Table 2 Variables list for modified mechanistic model based on a publication by Frahm B^1^.

| **Variable** | **Unit** | **Description** |
| --- | --- | --- |
| $c_{NH4}$ | mmol/L | Ammonia concentration in the bioreactor |
| $c_{GLC}$ | mmol/L | Glucose concentration in the bioreactor |
| $c_{GLN}$ | mmol/L | Glutamine concentration in the bioreactor |
| $c_{LAC}$ | mmol/L | Lactate concentration in the bioreactor |
| $c_{LIM_{medium}}$ | mmol/L | Concentration of limiting substrate in the bioreactor |
| $mu$ | 1/h | Cell-specific growth rate |
| $mu_{d}$ | 1/h | Cell-specific death rate |
| $F_{GLC}$ | L/h | Feed flow of glucose feed |
| $F_{GLN}$ | L/h | Feed flow of glutamine feed |
| $F_{medium}$ | L/h | Feed flow of medium feed/perfusion rate |
| $F_{out}$ | L/h | Permeate flow |
| $X_{v}$ | cells/L | Viable cell concentration in the bioreactor |
| $X_{t}$ | cells/L | Total cell concentration in the bioreactor |
| $q_{GLC}$ | mmol/(cell h) | Cell-specific glucose uptake rate |
| $q_{LAC}$ | mmol/(cell h) | Cell-specific lactate production rate |
| $q_{GLN}$ | mmol/(cell h) | Cell-specific glutamine uptake rate |
| $q_{mAb}$ | mmol/(cell h) | Cell-specific mAb production rate |
| $q_{LIM}$ | mmol/(cell h) | Cell-specific uptake rate of limiting substrate |
| $V$ | L | Reactor volume |
| $V_{f}$ | L | Cumulative feed volume |

^1^ Frahm, B., *Seed train optimization for cell culture*, in *Animal Cell Biotechnology*. 2014, Springer. p. 355- 367

Constraints

$\boldsymbol{LIMIT} \boldsymbol{c}_{\boldsymbol{NH}\boldsymbol{4}}\boldsymbol{\geq}\boldsymbol{0}$

$\boldsymbol{LIMIT} \boldsymbol{c}_{\boldsymbol{GLC}}\boldsymbol{\geq}\boldsymbol{0}$

$\boldsymbol{LIMIT} \boldsymbol{c}_{\boldsymbol{GLN}}\boldsymbol{\geq}\boldsymbol{0}$

$\boldsymbol{LIMIT} \boldsymbol{c}_{\boldsymbol{LAC}}\boldsymbol{\geq}\boldsymbol{0}$

Mass Balances

$\frac{d X_{v}}{dt}=\left( mu-mu_{d}-\frac{F_{GLC}+F_{GLN}+F_{medium}-F_{out}}{V} \right)*X{}_{v}$

$\frac{d X_{t}}{dt}=mu*X_{v}-\frac{F_{GLC}+F_{GLN}+F_{medium}-F_{out}}{V}*X_{t}-k_{lys}*\left( X_{t}-X_{v} \right)$

$\frac{d c_{GLC}}{dt}=\frac{F_{GLC}}{V}*c_{GLC_{F}}+\frac{F_{medium}}{V}*{c_{GLC}}_{medium}-\frac{F_{GLC}+F_{GLN}+F_{medium}-F_{out}}{V}*c{}_{GLC}-q_{GLC}*X_{V}-\frac{F_{out}}{V}*c_{GLC}$

$\frac{d c_{GLN}}{dt}=\frac{F_{GLN}}{V}*c_{GLN_{F}}+\frac{F_{medium}}{V}*{c_{GLN}}_{medium}-\frac{F_{GLC}+F_{GLN}+F_{medium}-F_{out}}{V}*c{}_{GLN}-q_{GLN}*X_{V}-\frac{F_{out}}{V}*c_{GLN}-{k_{GLN}}_{deg}* c{}_{GLN}$

$\frac{d c_{LAC}}{dt}=-\frac{F_{GLC}+F_{GLN}+F_{medium}-F_{out}}{V}*c_{LAC}+q_{LAC}*X_{V}-\frac{F_{out}}{V}*c_{LAC}$

$\frac{d c_{NH4}}{dt}=-\frac{F_{GLC}+F_{GLN}+F_{medium}-F_{out}}{V}*c_{NH4}+q_{NH4}*X_{V}-\frac{F_{out}}{V}*c_{NH4}$

$\frac{d c_{mAb}}{dt}=-\frac{F_{GLC}+F_{GLN}+F_{medium}-F_{out}}{V}*c_{mAb}+q_{mAb}*X_{V}-\frac{F_{out}}{V}*c_{mAb}$

$\frac{d c_{LIM}}{dt}=\frac{F_{medium}}{V}*{c_{LIM}}_{medium}-\frac{F_{GLC}+F_{GLN}+F_{medium}-F_{out}}{V}*c_{LIM}-q_{LIM}*X_{V}-\frac{F_{out}}{V}*c_{LIM}$

$\frac{dV}{dt}=F_{GLC}+F_{GLN}+F_{medium}-F_{out}$

$\frac{dV_{f}}{dt}=F_{medium}$

**Kinetics**

$mu={mu}_{max}*\frac{c_{GLC}}{c_{GLC}+{K_{S}}_{GLC}}*\frac{c_{GLN}}{c_{GLN}+{K_{S}}_{GLN}}*\frac{c_{LIM}}{c_{LIM}+{K_{S}}_{LIM}}$

$mu_{d}={mu}_{d_{min}}$

$q_{GLC}={q_{GLC}}_{max}*\frac{c_{GLC}}{c_{GLC}+k_{GLC}}$

$q_{GLN}={q_{GLN}}_{max}*\frac{c_{GLN}}{c_{GLN}+k_{GLN}}*K_{p_{GLN}}$

$q_{LAC}=Y_{LAC, GLC}*q_{GLC}$

$q_{NH4}=Y_{NH4, GLN}*q_{GLN}$

$q_{LIM}=q_{LIM_{max}}*\frac{c_{LIM}}{c_{LIM}+k_{LIM}}$

$q_{mAb}={q_{mAb}}_{max}$

$if c_{GLC}>{c_{GLC}}_{boarder}$ $K_{p_{GLN}}=1$

$else$ $K_{p_{GLN}}=1+alpha*{(c_{GLC}}_{border}-c_{GLC})$

$if time > 48 then$ $Y_{LAC, GLC}=Y_{LAC, GLC II}$

$else$ $Y_{LAC, GLC}=Y_{LAC, GLC I}$

$if time > 48 then$ $Y_{NH4, GLN}=Y_{NH4, GLN II}$

$\boldsymbol{else}$ $\boldsymbol{Y}_{\boldsymbol{NH}\boldsymbol{4, GLN}}\boldsymbol{=}\boldsymbol{Y}_{\boldsymbol{NH}\boldsymbol{4, GLN I}}$

**Transcriptome profiling using next-generation sequencing (NGS)**

Effect and network analysis were performed with QIAGEN Ingenuity Pathway Analysis (IPA) summer release 2016 (IPA®, QIAGEN, USA, <http://www.ingenuity.com/>). The analysis are based on literature findings compiled in the Ingenuity® Knowledge Base and use the *Z*-score algorithm to evaluate effects or make predictions. The *Z*-score algorithm design is described in detail by Krämer et al.².


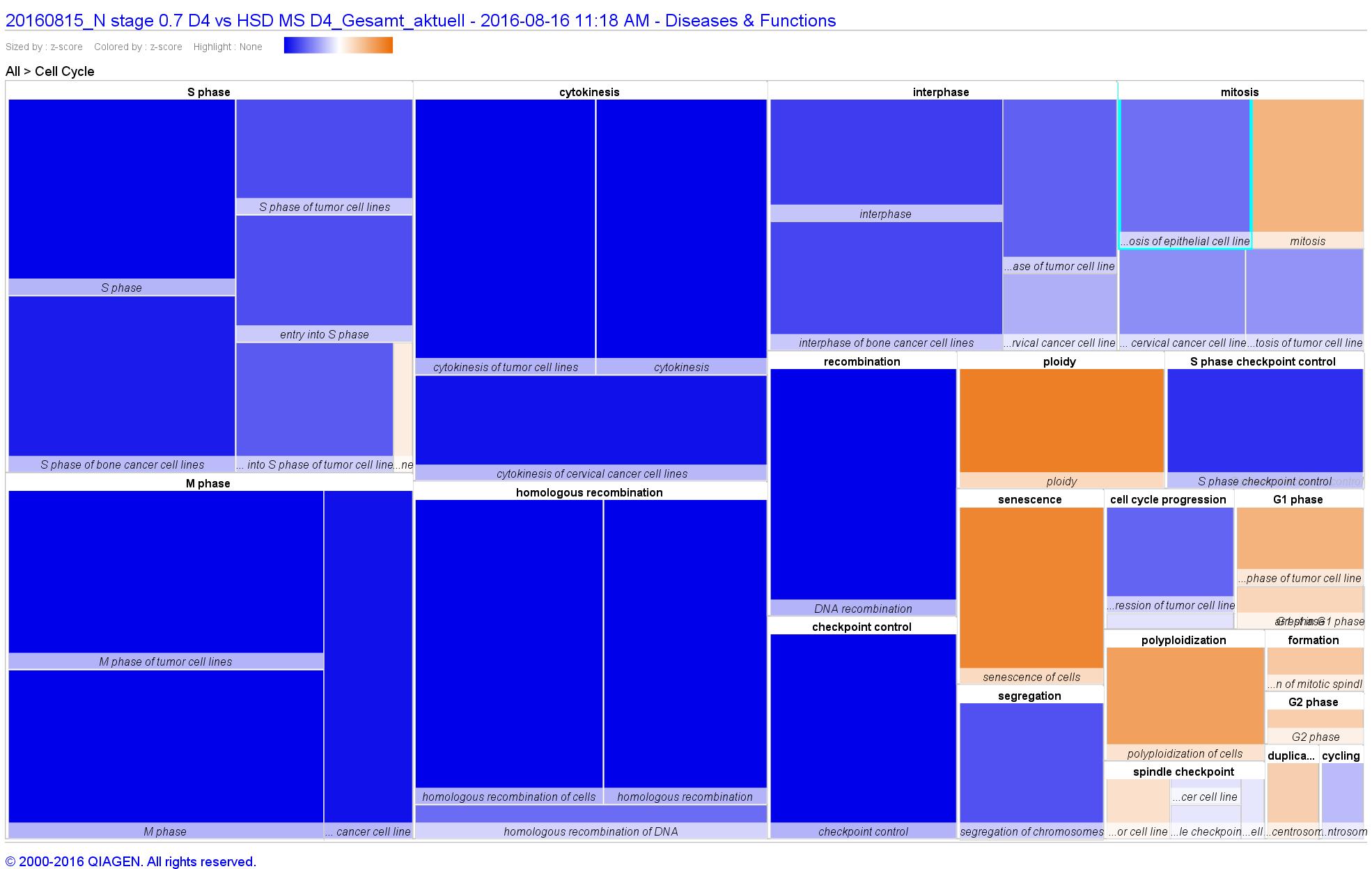


Supplementary Figure 3 Effect analysis for the function cell cycle: prognosis for regulation in final uHSD compared to control setup based on differentially expressed genes. Size and color intensity of the boxes comply with the *Z-score*. Z ≥2 (orange): function significantly increased, Z ≤ ‑2 (blue), significantly decreased.

² Krämer A, Green J, Pollard J Jr, Tugendreich S. *Causal analysis approaches in Ingenuity Pathway Analysis*. Bioinformatics. 2014;30(4):523–530. doi:10.1093/bioinformatics/btt703


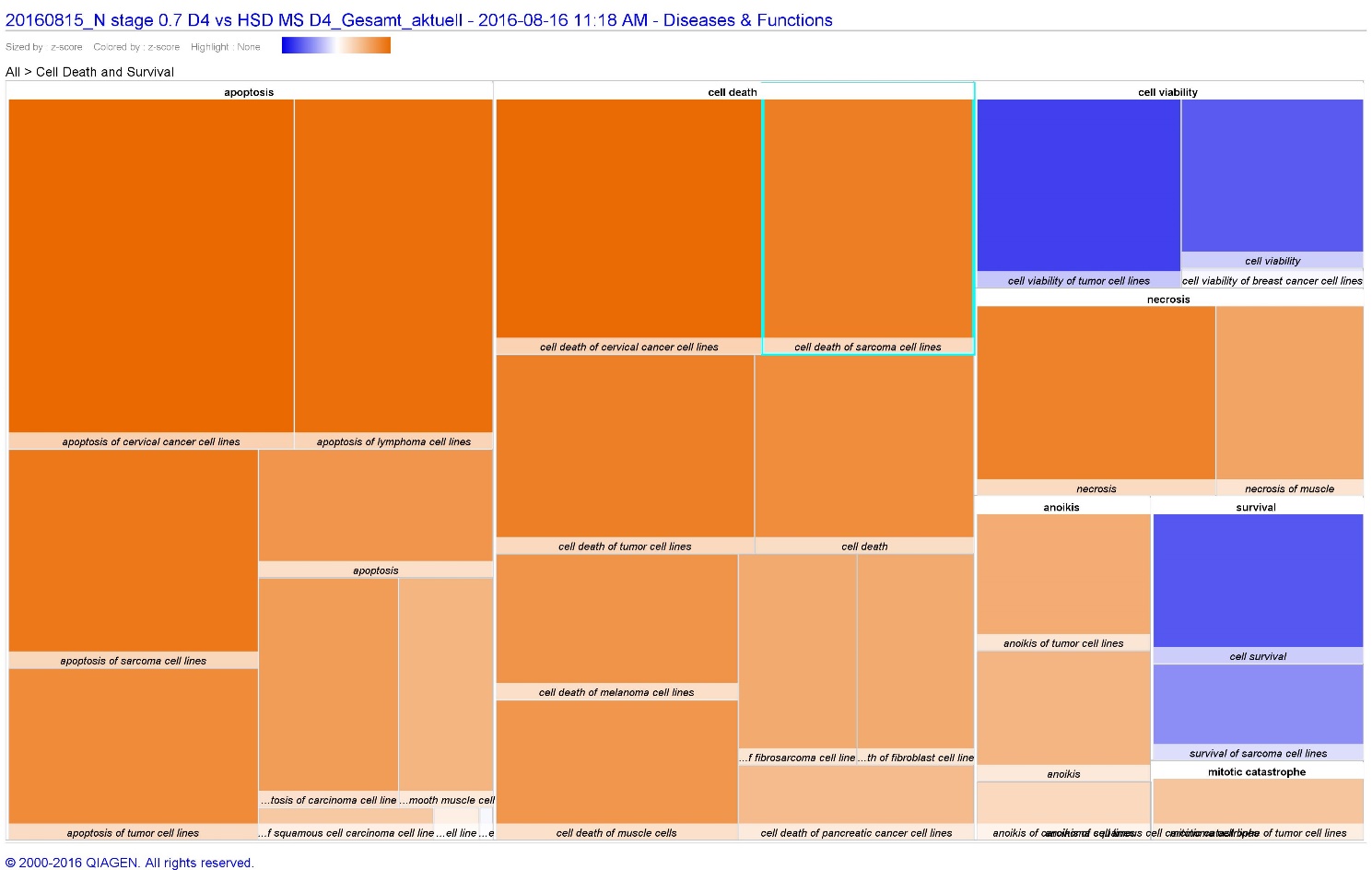


Supplementary Figure 4 Effect analysis for the function cell death and survival: prognosis for regulation in final uHSD compared to control setup based on differentially expressed genes. Size and color intensity of the boxes comply with the *Z-score*. Z ≥2 (orange): function significantly increased, Z ≤ ‑2 (blue), significantly decreased.


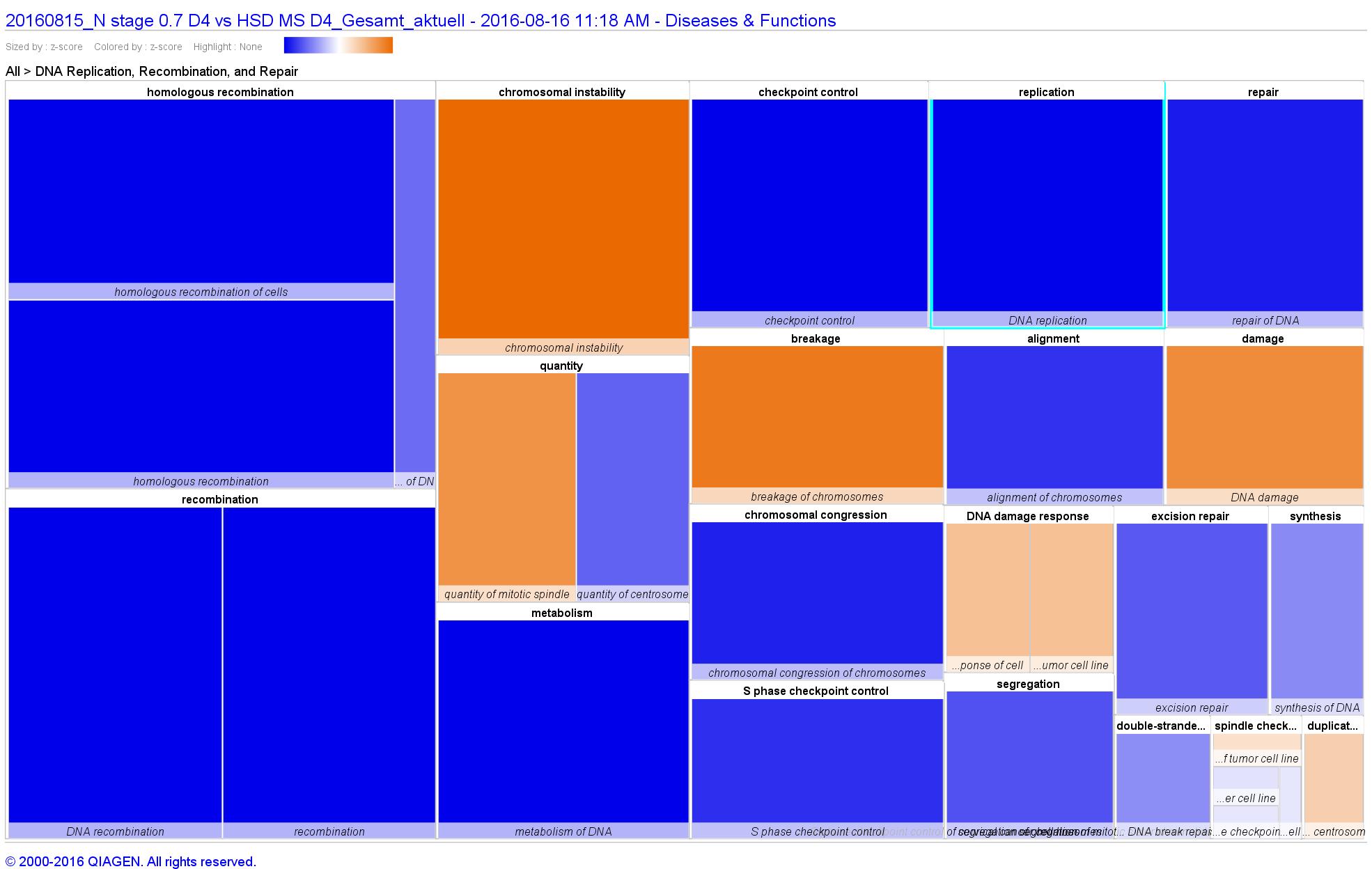


Supplementary Figure 5 Effect analysis for the function DNA replication, recombination and repair: prognosis for regulation in final uHSD compared to control setup based on differentially expressed genes. Size and color intensity of the boxes comply with the *z-score*. Z ≥2 (orange): function significantly increased, Z ≤ ‑2 (blue), significantly decreased.

**Supplementary Table 3 Fold changes for E2F1 and for down-regulated genes with E2F1 binding motives in their upstream DNA regions calculated in comparison to the reference sampling point control day 4 (yellow). Significantly down-regulated genes are highlighted in red, significantly up-regulated genes are highlighted in green.**

| ID | Fold changes [-] compared to control day 4 | | | | | | | | | | | |
| --- | --- | --- | --- | --- | --- | --- | --- | --- | --- | --- | --- | --- |
|  | control | | | | uHSD v0.1 | | | | uHSD | | | |
|  | day 0 | day 1 | day 4 | day 11 | day 0 | day 1 | day 4 | day 11 | day 0 | day 1 | day 4 | day 11 |
| E2F1 regulation compared to day 4 control | | | | | | | | | | | | |
| E2f1 | 1.0 | 1.5 | 1.0 | 0.5 | 1.0 | 0.9 | 0.4 | 0.4 | 0.9 | 0.9 | 0.4 | 0.5 |
| Down-regulated genes compared to control on day 4 with E2F1 binding motive | | | | | | | | | | | | |
| 4930579G24Rik | 0.7 | 1.2 | 1.0 | 0.3 | 0.6 | 1.2 | 0.4 | 0.5 | 0.6 | 1.2 | 0.3 | 0.5 |
| Abhd6 | 0.3 | 0.3 | 1.0 | 0.6 | 0.4 | 1.2 | 0.5 | 0.2 | 0.5 | 1.1 | 0.5 | 0.4 |
| Anp32e | 1.5 | 1.9 | 1.0 | 0.5 | 1.4 | 1.2 | 0.5 | 0.4 | 1.6 | 1.2 | 0.5 | 0.3 |
| Aspm | 1.0 | 1.4 | 1.0 | 0.2 | 0.7 | 0.8 | 0.1 | 0.2 | 0.7 | 0.9 | 0.2 | 0.1 |
| Atf5 | 1.1 | 0.9 | 1.0 | 0.4 | 1.5 | 0.8 | 0.3 | 0.3 | 1.7 | 0.7 | 0.2 | 0.7 |
| Bc030867 | 1.1 | 1.5 | 1.0 | 0.2 | 1.2 | 1.0 | 0.1 | 0.1 | 1.1 | 0.9 | 0.1 | 0.1 |
| Brca2 | 0.6 | 1.7 | 1.0 | 0.3 | 0.6 | 1.1 | 0.3 | 0.5 | 0.6 | 1.1 | 0.3 | 0.3 |
| Bub1 | 0.9 | 1.4 | 1.0 | 0.2 | 0.8 | 0.9 | 0.1 | 0.1 | 0.8 | 1.0 | 0.1 | 0.1 |
| C1qbp | 2.2 | 2.0 | 1.0 | 0.7 | 2.1 | 1.2 | 0.4 | 0.8 | 2.7 | 0.9 | 0.6 | 0.7 |
| Casc5 | 1.1 | 1.6 | 1.0 | 0.1 | 0.9 | 1.0 | 0.1 | 0.1 | 0.9 | 1.1 | 0.1 | 0.1 |
| Cbx5 | 1.2 | 1.7 | 1.0 | 0.4 | 1.2 | 1.1 | 0.3 | 0.3 | 1.2 | 1.1 | 0.4 | 0.3 |
| Ccdc150 | 1.1 | 1.2 | 1.0 | 0.3 | 1.1 | 0.9 | 0.3 | 0.5 | 1.0 | 1.1 | 0.3 | 0.5 |
| Ccdc165 | 0.5 | 1.0 | 1.0 | 0.6 | 0.6 | 1.2 | 0.5 | 0.7 | 0.5 | 0.9 | 0.5 | 0.7 |
| Ccnb2 | 1.1 | 1.4 | 1.0 | 0.1 | 1.0 | 0.7 | 0.0 | 0.0 | 0.9 | 0.9 | 0.1 | 0.0 |
| Cdca2 | 0.7 | 1.3 | 1.0 | 0.1 | 0.5 | 0.9 | 0.1 | 0.1 | 0.5 | 1.0 | 0.1 | 0.1 |
| Cenpl | 0.8 | 1.4 | 1.0 | 0.2 | 0.6 | 0.9 | 0.2 | 0.3 | 0.6 | 1.2 | 0.2 | 0.3 |
| Cenpm | 1.0 | 1.5 | 1.0 | 0.5 | 1.0 | 1.0 | 0.4 | 0.4 | 0.8 | 0.9 | 0.5 | 0.5 |
| Cep44 | 1.2 | 1.4 | 1.0 | 0.5 | 1.0 | 1.0 | 0.4 | 0.8 | 1.1 | 1.3 | 0.5 | 0.7 |
| Cep55 | 1.2 | 1.2 | 1.0 | 0.1 | 1.0 | 0.9 | 0.1 | 0.1 | 1.0 | 1.0 | 0.1 | 0.1 |
| Cit | 0.6 | 1.3 | 1.0 | 0.2 | 0.5 | 0.9 | 0.1 | 0.2 | 0.5 | 0.9 | 0.2 | 0.1 |
| Comtd1 | 2.0 | 1.7 | 1.0 | 0.6 | 2.4 | 1.3 | 0.2 | 0.2 | 1.8 | 0.7 | 0.3 | 0.4 |
| Dlgap5 | 0.9 | 1.5 | 1.0 | 0.1 | 0.8 | 0.9 | 0.1 | 0.0 | 0.7 | 1.0 | 0.1 | 0.0 |
| Dot1l | 3.2 | 1.0 | 1.0 | 0.6 | 2.6 | 1.0 | 0.4 | 0.5 | 2.3 | 0.8 | 0.4 | 0.6 |
| Evi2a | 2.4 | 1.2 | 1.0 | 0.5 | 2.2 | 0.8 | 0.2 | 0.4 | 2.4 | 1.0 | 0.3 | 0.4 |
| Exo1 | 1.1 | 1.9 | 1.0 | 0.1 | 1.1 | 1.2 | 0.1 | 0.0 | 1.1 | 1.1 | 0.1 | 0.0 |
| Exosc2 | 1.5 | 1.6 | 1.0 | 0.5 | 1.5 | 1.1 | 0.4 | 0.4 | 1.4 | 0.9 | 0.5 | 0.5 |
| Fancd2 | 1.0 | 1.6 | 1.0 | 0.2 | 0.9 | 1.1 | 0.1 | 0.2 | 0.9 | 1.1 | 0.2 | 0.2 |
| Fanci | 1.0 | 1.9 | 1.0 | 0.2 | 0.9 | 1.0 | 0.1 | 0.3 | 1.1 | 1.2 | 0.2 | 0.2 |
| Fignl1 | 0.9 | 1.9 | 1.0 | 0.2 | 1.0 | 1.1 | 0.2 | 0.2 | 0.9 | 1.1 | 0.2 | 0.2 |
| Gene426_path1 | 0.8 | 0.9 | 1.0 | 0.5 | 0.6 | 0.8 | 0.3 | 0.2 | 0.8 | 0.9 | 0.4 | 0.3 |
| Gene1522_path1 | 0.8 | 1.0 | 1.0 | 0.7 | 1.0 | 0.9 | 0.5 | 0.5 | 0.7 | 1.1 | 0.7 | 0.6 |
| Gene1662_path1 | 1.5 | 1.6 | 1.0 | 0.6 | 1.6 | 1.2 | 0.5 | 0.5 | 1.7 | 1.1 | 0.6 | 0.4 |
| Gene3826_path1 | 1.1 | 1.0 | 1.0 | 0.7 | 1.1 | 0.9 | 0.4 | 0.3 | 0.9 | 0.8 | 0.5 | 0.6 |
| Gene4372_path1 | 1.3 | 1.2 | 1.0 | 0.6 | 1.4 | 1.1 | 0.1 | 0.3 | 1.0 | 0.9 | 0.5 | 0.3 |
| Gene4906_path1 | 1.6 | 1.5 | 1.0 | 0.6 | 1.6 | 0.9 | 0.5 | 0.4 | 1.5 | 1.0 | 0.4 | 0.4 |
| Gene5574_path1 | 1.6 | 2.1 | 1.0 | 0.3 | 1.9 | 1.1 | 0.3 | 0.2 | 1.5 | 1.0 | 0.4 | 0.2 |
| Gene7291_path1 | 0.9 | 1.2 | 1.0 | 0.3 | 0.9 | 0.7 | 0.3 | 0.3 | 0.8 | 0.9 | 0.4 | 0.4 |
| Gene7943_path1 | 1.4 | 1.6 | 1.0 | 0.4 | 1.4 | 1.0 | 0.5 | 0.5 | 1.5 | 1.1 | 0.6 | 0.5 |
| Gene10260_path1 | 1.5 | 1.5 | 1.0 | 0.3 | 1.2 | 0.8 | 0.2 | 0.2 | 1.1 | 0.9 | 0.3 | 0.2 |
| Gene10383_path1 | 1.1 | 1.1 | 1.0 | 0.8 | 1.3 | 1.0 | 0.4 | 0.3 | 0.9 | 0.7 | 0.5 | 0.5 |
| Gene11326_path1 | 1.3 | 1.3 | 1.0 | 0.7 | 1.3 | 0.8 | 0.4 | 0.3 | 1.0 | 0.7 | 0.6 | 0.6 |
| Gene11671_path1 | 1.8 | 1.2 | 1.0 | 0.5 | 1.8 | 0.9 | 0.4 | 0.4 | 1.6 | 1.0 | 0.4 | 0.3 |
| Gene12697_path1 | 1.0 | 1.2 | 1.0 | 0.7 | 1.3 | 0.9 | 0.4 | 0.3 | 1.0 | 0.8 | 0.6 | 0.4 |
| Gene12835_path1 | 0.7 | 1.4 | 1.0 | 0.4 | 0.8 | 1.0 | 0.3 | 0.1 | 0.6 | 0.7 | 0.3 | 0.2 |
| Gene13276_path1 | 1.1 | 1.1 | 1.0 | 0.7 | 1.2 | 1.1 | 0.3 | 0.4 | 0.9 | 0.5 | 0.3 | 0.6 |
| Gene14747_path1 | 2.0 | 1.6 | 1.0 | 0.6 | 2.1 | 1.3 | 0.4 | 0.3 | 1.8 | 0.9 | 0.5 | 0.2 |
| Gene16724_path1 | 0.6 | 1.1 | 1.0 | 0.7 | 0.9 | 0.8 | 0.5 | 1.0 | 0.8 | 0.7 | 0.7 | 0.8 |
| Gene16897_path1 | 1.4 | 0.9 | 1.0 | 0.7 | 1.5 | 0.9 | 0.3 | 0.4 | 0.9 | 0.4 | 0.3 | 0.5 |
| Gene17218_path1 | 1.0 | 0.9 | 1.0 | 0.6 | 1.0 | 0.9 | 0.4 | 0.3 | 1.0 | 0.8 | 0.5 | 0.3 |
| Gene18238_path1 | 0.6 | 0.5 | 1.0 | 0.7 | 0.8 | 1.1 | 0.4 | 0.7 | 0.4 | 0.7 | 0.4 | 0.8 |
| Gene21046_path1 | 1.5 | 1.6 | 1.0 | 0.5 | 1.6 | 1.1 | 0.3 | 0.4 | 1.5 | 1.0 | 0.4 | 0.4 |
| Gene21315_path1 | 1.8 | 1.5 | 1.0 | 0.6 | 1.9 | 1.0 | 0.5 | 0.5 | 1.8 | 0.8 | 0.5 | 0.6 |
| Gene23169_path1 | 1.9 | 1.7 | 1.0 | 0.5 | 2.2 | 1.2 | 0.5 | 0.3 | 2.2 | 1.1 | 0.4 | 0.2 |
| Gtpbp2 | 0.4 | 0.3 | 1.0 | 0.6 | 0.3 | 1.1 | 0.5 | 0.7 | 0.3 | 1.1 | 0.5 | 0.6 |
| G_667 | 1.0 | 1.1 | 1.0 | 0.7 | 1.1 | 0.9 | 0.5 | 0.3 | 1.0 | 0.9 | 0.6 | 0.5 |
| G_864 | 1.7 | 1.4 | 1.0 | 0.5 | 1.5 | 0.9 | 0.4 | 0.4 | 1.7 | 1.0 | 0.4 | 0.3 |
| G_2008 | 1.6 | 1.4 | 1.0 | 0.6 | 1.4 | 1.1 | 0.4 | 0.6 | 1.4 | 1.1 | 0.3 | 0.4 |
| G_3151 | 1.2 | 1.2 | 1.0 | 0.4 | 1.0 | 1.0 | 0.2 | 0.5 | 1.1 | 1.2 | 0.5 | 0.5 |
| G_3246 | 1.7 | 1.6 | 1.0 | 0.5 | 2.0 | 1.2 | 0.4 | 0.3 | 1.8 | 1.1 | 0.4 | 0.2 |
| G_3929 | 0.7 | 0.6 | 1.0 | 0.6 | 0.7 | 1.6 | 0.5 | 0.3 | 0.7 | 1.4 | 0.5 | 0.3 |
| G_5129 | 0.9 | 1.0 | 1.0 | 0.5 | 0.5 | 1.1 | 0.5 | 0.4 | 0.8 | 0.6 | 0.6 | 0.6 |
| G_5971 | 1.7 | 2.9 | 1.0 | 0.4 | 2.3 | 1.0 | 0.1 | 0.3 | 1.8 | 1.2 | 0.7 | 0.8 |
| G_6826 | 1.9 | 1.5 | 1.0 | 0.6 | 1.9 | 1.1 | 0.4 | 0.1 | 1.6 | 0.8 | 0.4 | 0.2 |
| G_7735 | 1.1 | 2.0 | 1.0 | 0.4 | 1.2 | 1.1 | 0.3 | 0.5 | 0.8 | 0.5 | 0.3 | 0.7 |
| G_8419 | 1.6 | 1.7 | 1.0 | 0.6 | 1.7 | 1.0 | 0.5 | 0.4 | 1.5 | 0.9 | 0.5 | 0.4 |
| G_8892 | 0.8 | 1.0 | 1.0 | 0.3 | 0.9 | 1.0 | 0.2 | 0.2 | 0.7 | 0.8 | 0.2 | 0.3 |
| G_9255 | 1.1 | 1.8 | 1.0 | 0.5 | 1.4 | 1.3 | 0.4 | 0.3 | 1.4 | 1.1 | 0.4 | 0.5 |
| G_9393 | 2.0 | 1.3 | 1.0 | 0.6 | 1.7 | 1.2 | 0.5 | 0.5 | 1.3 | 0.7 | 0.5 | 0.4 |
| G_9498 | 1.2 | 1.7 | 1.0 | 0.2 | 1.0 | 1.1 | 0.1 | 0.1 | 0.9 | 1.0 | 0.1 | 0.1 |
| G_9788 | 1.1 | 1.7 | 1.0 | 0.5 | 1.0 | 1.0 | 0.4 | 0.3 | 1.0 | 1.0 | 0.4 | 0.3 |
| G_9800 | 1.0 | 1.5 | 1.0 | 0.2 | 0.8 | 1.0 | 0.1 | 0.1 | 0.7 | 1.0 | 0.1 | 0.1 |
| G_10829 | 0.7 | 1.1 | 1.0 | 0.4 | 0.6 | 0.9 | 0.4 | 0.5 | 0.7 | 1.0 | 0.4 | 0.4 |
| Haus8 | 1.0 | 1.7 | 1.0 | 0.4 | 1.1 | 1.0 | 0.3 | 0.2 | 1.0 | 1.0 | 0.4 | 0.3 |
| Hdgf | 1.2 | 1.6 | 1.0 | 0.5 | 1.2 | 1.0 | 0.4 | 0.4 | 1.2 | 0.9 | 0.4 | 0.4 |
| Hirip3 | 1.1 | 2.2 | 1.0 | 0.3 | 1.2 | 1.1 | 0.2 | 0.4 | 1.1 | 1.1 | 0.3 | 0.4 |
| Hjurp | 1.1 | 1.3 | 1.0 | 0.2 | 0.9 | 0.9 | 0.2 | 0.2 | 0.8 | 0.9 | 0.2 | 0.1 |
| Loc361346 | 0.9 | 1.3 | 1.0 | 0.4 | 0.8 | 0.9 | 0.3 | 0.5 | 0.8 | 1.0 | 0.4 | 0.4 |
| Loc690976 | 1.1 | 1.3 | 1.0 | 0.7 | 1.0 | 1.1 | 0.4 | 0.2 | 0.9 | 0.8 | 0.5 | 0.3 |
| Loc100310874 | 0.7 | 1.1 | 1.0 | 0.1 | 0.5 | 0.8 | 0.1 | 0.0 | 0.5 | 1.0 | 0.1 | 0.0 |
| Lsm3 | 1.4 | 1.9 | 1.0 | 0.4 | 1.3 | 0.9 | 0.3 | 0.4 | 1.3 | 1.0 | 0.4 | 0.3 |
| Matn4 | 0.7 | 1.0 | 1.0 | 0.2 | 0.8 | 1.3 | 0.1 | 0.1 | 0.6 | 0.7 | 0.0 | 0.1 |
| Mbd3 | 2.1 | 1.4 | 1.0 | 0.6 | 2.0 | 1.0 | 0.4 | 0.7 | 1.6 | 0.7 | 0.5 | 1.0 |
| Mcm4 | 1.7 | 2.6 | 1.0 | 0.2 | 1.7 | 1.2 | 0.2 | 0.2 | 1.7 | 1.1 | 0.2 | 0.2 |
| Mcm5 | 1.7 | 2.3 | 1.0 | 0.1 | 1.5 | 1.1 | 0.1 | 0.1 | 1.4 | 1.0 | 0.1 | 0.1 |
| Mcm8 | 1.0 | 1.9 | 1.0 | 0.4 | 1.0 | 1.0 | 0.4 | 0.5 | 1.0 | 0.9 | 0.4 | 0.4 |
| Mybl1 | 0.4 | 1.2 | 1.0 | 0.2 | 0.4 | 1.0 | 0.2 | 0.4 | 0.4 | 1.0 | 0.2 | 0.3 |
| Myo1g | 0.2 | 0.3 | 1.0 | 0.6 | 0.2 | 0.6 | 0.3 | 0.1 | 0.2 | 0.5 | 0.4 | 0.3 |
| Ncapg | 0.9 | 1.3 | 1.0 | 0.2 | 0.8 | 1.0 | 0.2 | 0.2 | 0.8 | 1.1 | 0.2 | 0.2 |
| Ndp | 4.9 | 1.4 | 1.0 | 0.3 | 4.3 | 1.3 | 0.3 | 0.2 | 4.6 | 1.2 | 0.3 | 0.1 |
| Nucks1 | 1.6 | 1.4 | 1.0 | 0.4 | 1.6 | 1.0 | 0.4 | 0.3 | 1.5 | 1.0 | 0.4 | 0.3 |
| Paics | 1.1 | 1.4 | 1.0 | 0.5 | 1.2 | 1.1 | 0.5 | 0.4 | 1.2 | 1.0 | 0.4 | 0.4 |
| Polr3k | 0.8 | 1.2 | 1.0 | 0.8 | 0.7 | 0.8 | 0.5 | 0.6 | 0.9 | 0.8 | 0.6 | 0.8 |
| Rad51l1 | 1.0 | 1.4 | 1.0 | 0.4 | 0.9 | 1.0 | 0.5 | 0.6 | 0.9 | 1.1 | 0.4 | 0.6 |
| Rad54l | 0.8 | 1.7 | 1.0 | 0.1 | 0.8 | 1.0 | 0.1 | 0.1 | 0.8 | 1.0 | 0.1 | 0.1 |
| Rcbtb2 | 1.2 | 1.9 | 1.0 | 0.5 | 1.3 | 1.0 | 0.4 | 0.3 | 1.1 | 1.0 | 0.4 | 0.3 |
| Rfc3 | 1.1 | 1.9 | 1.0 | 0.4 | 1.0 | 1.2 | 0.4 | 0.3 | 1.1 | 1.0 | 0.4 | 0.3 |
| Rfc5 | 1.4 | 1.8 | 1.0 | 0.3 | 1.4 | 1.0 | 0.2 | 0.2 | 1.4 | 1.1 | 0.3 | 0.2 |
| Rgd1304563 | 1.2 | 0.9 | 1.0 | 0.7 | 1.2 | 1.0 | 0.5 | 0.2 | 1.0 | 0.7 | 0.4 | 0.3 |
| Rgd1308541 | 1.1 | 1.8 | 1.0 | 0.2 | 0.9 | 1.0 | 0.1 | 0.1 | 0.9 | 1.1 | 0.2 | 0.1 |
| Rin2 | 1.6 | 1.0 | 1.0 | 0.6 | 1.4 | 1.0 | 0.4 | 0.3 | 1.6 | 1.0 | 0.4 | 0.3 |
| Rna961_path1 | 0.6 | 1.1 | 1.0 | 0.3 | 0.6 | 1.3 | 0.4 | 0.3 | 0.6 | 1.1 | 0.3 | 0.4 |
| Rna2084_path1 | 2.1 | 1.8 | 1.0 | 0.4 | 2.3 | 1.0 | 0.3 | 0.2 | 2.3 | 1.0 | 0.3 | 0.3 |
| Rna2153_path1 | 1.9 | 1.7 | 1.0 | 0.5 | 2.2 | 1.0 | 0.5 | 0.5 | 2.2 | 0.9 | 0.4 | 0.5 |
| Rna3107_path1 | 2.3 | 1.0 | 1.0 | 0.6 | 1.9 | 1.1 | 0.5 | 0.6 | 1.9 | 1.0 | 0.4 | 0.5 |
| Rna3324_path1 | 0.8 | 1.2 | 1.0 | 0.1 | 0.5 | 0.9 | 0.1 | 0.0 | 0.5 | 1.0 | 0.1 | 0.0 |
| Rna3326_path1 | 0.5 | 1.4 | 1.0 | 0.1 | 0.5 | 0.9 | 0.1 | 0.1 | 0.4 | 1.1 | 0.1 | 0.1 |
| Rna4486_path1 | 1.7 | 1.9 | 1.0 | 0.4 | 1.8 | 1.0 | 0.3 | 0.4 | 1.7 | 1.0 | 0.4 | 0.5 |
| Rna4510_path1 | 1.3 | 1.1 | 1.0 | 0.8 | 1.4 | 0.9 | 0.4 | 0.6 | 1.1 | 0.6 | 0.5 | 1.1 |
| Rna5401_path1 | 1.1 | 1.2 | 1.0 | 0.1 | 1.0 | 0.7 | 0.1 | 0.0 | 1.0 | 0.8 | 0.1 | 0.0 |
| Rna6607_path1 | 1.6 | 1.4 | 1.0 | 0.4 | 1.7 | 1.0 | 0.4 | 0.3 | 1.6 | 1.1 | 0.4 | 0.3 |
| Rna7176_path1 | 0.8 | 2.2 | 1.0 | 0.2 | 1.0 | 1.0 | 0.2 | 0.2 | 0.9 | 0.9 | 0.2 | 0.2 |
| Rna7757_path2 | 0.5 | 1.1 | 1.0 | 0.5 | 0.6 | 1.1 | 0.5 | 0.5 | 0.6 | 1.0 | 0.4 | 0.6 |
| Rna8829_path1 | 0.3 | 0.8 | 1.0 | 0.2 | 0.3 | 0.8 | 0.4 | 0.4 | 0.4 | 0.7 | 0.5 | 0.3 |
| Rna9686_path1 | 1.1 | 1.5 | 1.0 | 0.3 | 1.0 | 1.1 | 0.3 | 0.4 | 1.1 | 1.1 | 0.4 | 0.4 |
| Rna10254_path1 | 0.6 | 0.8 | 1.0 | 0.7 | 0.7 | 1.0 | 0.4 | 0.6 | 0.6 | 0.8 | 0.5 | 0.8 |
| Rna11623_path1 | 0.8 | 1.1 | 1.0 | 0.6 | 1.0 | 1.0 | 0.3 | 0.4 | 1.0 | 0.9 | 0.4 | 0.4 |
| Rna14692_path1 | 0.9 | 1.2 | 1.0 | 0.6 | 1.0 | 1.0 | 0.4 | 0.3 | 0.9 | 0.9 | 0.6 | 0.5 |
| Rna15598_path1 | 0.7 | 1.5 | 1.0 | 0.2 | 0.8 | 1.3 | 0.2 | 0.1 | 0.9 | 1.3 | 0.2 | 0.1 |
| Rna16447_path1 | 1.0 | 2.4 | 1.0 | 0.2 | 1.0 | 1.3 | 0.2 | 0.2 | 1.0 | 1.2 | 0.2 | 0.2 |
| Rna17982_path1 | 2.0 | 0.8 | 1.0 | 0.8 | 1.0 | 0.8 | 0.4 | 0.8 | 1.0 | 0.8 | 0.4 | 0.9 |
| Rna18684_path1 | 1.8 | 1.4 | 1.0 | 0.6 | 1.7 | 0.9 | 0.4 | 0.3 | 1.5 | 0.8 | 0.4 | 0.4 |
| Rna19460_path1 | 1.5 | 2.1 | 1.0 | 0.4 | 1.4 | 0.8 | 0.4 | 0.4 | 1.2 | 0.8 | 0.3 | 0.4 |
| Rna19812_path1 | 0.8 | 1.7 | 1.0 | 0.3 | 0.8 | 1.1 | 0.4 | 0.5 | 0.9 | 1.1 | 0.4 | 0.4 |
| Rna20610_path1 | 1.0 | 1.1 | 1.0 | 0.2 | 0.8 | 0.9 | 0.2 | 0.2 | 0.8 | 1.0 | 0.2 | 0.2 |
| Rna20822_path1 | 1.1 | 1.4 | 1.0 | 0.4 | 1.3 | 1.0 | 0.4 | 0.2 | 1.3 | 1.0 | 0.4 | 0.2 |
| Rna22089_path1 | 1.1 | 1.0 | 1.0 | 0.5 | 1.2 | 0.9 | 0.2 | 0.2 | 0.8 | 0.3 | 0.2 | 0.3 |
| Rnaseh2a | 1.4 | 2.3 | 1.0 | 0.2 | 1.4 | 1.0 | 0.2 | 0.2 | 1.3 | 0.9 | 0.2 | 0.3 |
| Rnaseh2b | 1.4 | 1.5 | 1.0 | 0.5 | 1.3 | 1.0 | 0.5 | 0.5 | 1.3 | 0.9 | 0.4 | 0.4 |
| Rrm1 | 1.4 | 2.0 | 1.0 | 0.3 | 1.3 | 1.0 | 0.2 | 0.3 | 1.2 | 1.1 | 0.3 | 0.3 |
| S100a3 | 0.2 | 0.6 | 1.0 | 0.8 | 0.2 | 0.9 | 0.4 | 0.2 | 0.2 | 0.8 | 0.6 | 0.3 |
| S100a16 | 0.7 | 0.9 | 1.0 | 0.6 | 0.8 | 1.2 | 0.5 | 0.3 | 0.8 | 1.0 | 0.6 | 0.5 |
| Sirt1 | 1.2 | 1.3 | 1.0 | 0.5 | 1.1 | 1.2 | 0.5 | 0.6 | 1.2 | 1.2 | 0.5 | 0.5 |
| Smarcb1 | 1.0 | 1.2 | 1.0 | 0.6 | 1.0 | 1.1 | 0.5 | 0.4 | 1.0 | 0.9 | 0.4 | 0.4 |
| Sumo2 | 1.2 | 1.5 | 1.0 | 0.5 | 1.3 | 1.0 | 0.5 | 0.6 | 1.3 | 0.8 | 0.4 | 0.6 |
| Tlr2 | 1.5 | 0.9 | 1.0 | 0.4 | 1.7 | 1.1 | 0.3 | 0.2 | 2.1 | 1.3 | 0.3 | 0.2 |
| Tmem194 | 0.8 | 1.4 | 1.0 | 0.5 | 1.0 | 1.0 | 0.4 | 0.3 | 1.0 | 1.1 | 0.4 | 0.2 |
| Urm1 | 1.6 | 1.1 | 1.0 | 0.8 | 1.3 | 1.0 | 0.5 | 0.4 | 1.3 | 1.0 | 0.6 | 0.6 |
| Usp1 | 1.2 | 1.6 | 1.0 | 0.3 | 1.2 | 1.1 | 0.3 | 0.3 | 1.2 | 1.2 | 0.3 | 0.3 |
| Wdr62 | 0.9 | 1.0 | 1.0 | 0.2 | 0.8 | 1.0 | 0.1 | 0.2 | 0.7 | 0.9 | 0.1 | 0.2 |
| Zwint | 0.8 | 1.4 | 1.0 | 0.1 | 0.8 | 1.0 | 0.1 | 0.0 | 0.8 | 1.0 | 0.1 | 0.1 |

**Supplementary Table 4 IPA Upstream Regulator Analysis found 160 target genes of p53 in the investigated dataset. The analysis is based on expected causal effects between upstream regulators and their targets compiled in the Ingenuity® Knowledge Base (number of literature findings in parenthesis). Comparison of the targets’ actual direction of change to expectations derived from the literature, then issues a prediction for each upstream regulator. Overall, p53 is predicted to be activated (*Z-score* = 4.933, p-value= 2.99E-45).**

| ID | Genes in dataset | Expr Fold Change* | Causal effect between p53 and targets derived from Ingenuity® Knowledge Base | Individual prediction for p53 (based on measurement direction and expected causal effect for each target ) |
| --- | --- | --- | --- | --- |
| Col1a1 | COL1A1 | 114.441 | Upregulates (5) | Activated |
| Ptch1 | PTCH1 | 12.158 | Upregulates (1) | Activated |
| Ptger1 | PTGER1 | 10.061 | Regulates (1) | Affected |
| Ctgf | CCN2 | 8.345 | Regulates (3) | Affected |
| Pltp | PLTP | 8.311 | Upregulates (1) | Activated |
| Mb | MB | 8.275 | Regulates (1) | Affected |
| Fn1 | FN1 | 7.646 | Downregulates (4) | Inhibited |
| Mmp2 | MMP2 | 7.492 | Regulates (23) | Affected |
| Rgs16 | RGS16 | 5.638 | Upregulates (2) | Activated |
| Cp | CP | 5.461 | Upregulates (1) | Activated |
| Notch1 | NOTCH1 | 5.101 | Upregulates (21) | Activated |
| Fmo1 | FMO1 | 5.094 | Downregulates (1) | Inhibited |
| Trp53inp1 | TP53INP1 | 4.733 | Upregulates (13) | Activated |
| Ccl2 | Ccl2 | 4.477 | Downregulates (1) | Inhibited |
| Spp1 | SPP1 | 4.259 | Upregulates (3) | Activated |
| Thbs2 | THBS2 | 4.036 | Upregulates (1) | Activated |
| Bcl2l11 | BCL2L11 | 4.01 | Downregulates (8) | Inhibited |
| Fabp4 | FABP4 | 3.866 | Upregulates (2) | Activated |
| Timp2 | TIMP2 | 3.533 | Downregulates (1) | Inhibited |
| Ctsk | CTSK | 3.339 | Upregulates (1) | Activated |
| Serpine1 | SERPINE1 | 3.294 | Upregulates (21) | Activated |
| Pik3r3 | PIK3R3 | 3.219 | Regulates (3) | Affected |
| Bhlhe40 | BHLHE40 | 3.209 | Upregulates (8) | Activated |
| Pdgfrb | PDGFRB | 3.151 | Upregulates (3) | Activated |
| Bmf | BMF | 3.135 | Downregulates (2) | Inhibited |
| Tbxas1 | TBXAS1 | 3.08 | Downregulates (3) | Inhibited |
| Postn | POSTN | 3.049 | Regulates (5) | Affected |
| Tgm2 | TGM2 | 3.049 | Upregulates (1) | Activated |
| Akt3 | AKT3 | 2.888 | Downregulates (1) | Inhibited |
| Alb | ALB | 2.695 | Downregulates (2) | Inhibited |
| Gh | CSHL1 | 2.622 | Downregulates (1) | Inhibited |
| Ckmt1 | CKMT1A/CKMT1B | 2.6 | Regulates (2) | Affected |
| Rnase4 | RNASE4 | 2.592 | Regulates (1) | Affected |
| Sspn | SSPN | 2.554 | Regulates (1) | Affected |
| Clu | CLU | 2.517 | Upregulates (3) | Activated |
| Tnfsf10 | TNFSF10 | 2.48 | Upregulates (3) | Activated |
| Ccng2 | CCNG2 | 2.451 | Upregulates (2) | Activated |
| Ctsb | CTSB | 2.429 | Downregulates (1) | Inhibited |
| Tdo2 | TDO2 | 2.426 | Downregulates (1) | Inhibited |
| Tcn2 | TCN2 | 2.399 | Regulates (1) | Affected |
| Ifng | IFNG | 2.391 | Downregulates (5) | Inhibited |
| Pdgfra | PDGFRA | 2.382 | Upregulates (2) | Activated |
| Fstl1 | FSTL1 | 2.279 | Upregulates (1) | Activated |
| Nox4 | NOX4 | 2.166 | Regulates (1) | Affected |
| Ddr1 | DDR1 | 2.094 | Upregulates (2) | Activated |
| Tgfb3 | TGFB3 | 2.093 | Downregulates (1) | Inhibited |
| Chst12 | CHST12 | 2.085 | Upregulates (3) | Activated |
| Rhob | RHOB | 2.079 | Upregulates (2) | Activated |
| Ptgs2 | PTGS2 | 2.076 | Downregulates (23) | Inhibited |
| Dkk3 | DKK3 | 2.071 | Upregulates (1) | Activated |
| Lpin1 | LPIN1 | 2.049 | Upregulates (10) | Activated |
| Myo10 | MYO10 | 2.041 | Regulates (1) | Affected |
| Ubl3 | UBL3 | 2.02 | Regulates (1) | Affected |
| Bmp1 | BMP1 | 1.967 | Upregulates (2) | Activated |
| Il10 | IL10 | -2.004 | Regulates (2) | Affected |
| Ywhah | YWHAH | -2.006 | Upregulates (1) | Inhibited |
| Cdkn1a | CDKN1A | -2.03 | Upregulates (912) | Inhibited |
| Caml | CAMLG | -2.062 | Regulates (2) | Affected |
| Smarcb1 | SMARCB1 | -2.098 | Upregulates (1) | Inhibited |
| Sirt1 | SIRT1 | -2.114 | Downregulates (8) | Activated |
| Cdc25a | CDC25A | -2.12 | Downregulates (6) | Activated |
| Umps | UMPS | -2.142 | Downregulates (1) | Activated |
| Myo1c | MYO1C | -2.143 | Upregulates (1) | Inhibited |
| Rprm | RPRM | -2.154 | Upregulates (6) | Inhibited |
| Vim | VIM | -2.16 | Upregulates (6) | Inhibited |
| Ercc1 | ERCC1 | -2.205 | Upregulates (2) | Inhibited |
| Ezh2 | EZH2 | -2.208 | Downregulates (3) | Activated |
| Dhfr | DHFR | -2.238 | Downregulates (3) | Activated |
| Tap2 | TAP2 | -2.246 | Downregulates (1) | Activated |
| Mvd | MVD | -2.281 | Regulates (1) | Affected |
| Srsf3 | SRSF3 | -2.322 | Downregulates (1) | Activated |
| Wee1 | WEE1 | -2.35 | Downregulates (1) | Activated |
| E2f1 | E2F1 | -2.427 | Downregulates (8) | Activated |
| Wdhd1 | WDHD1 | -2.449 | Downregulates (2) | Activated |
| Fhl1 | FHL1 | -2.455 | Upregulates (3) | Inhibited |
| Mcm2 | MCM2 | -2.463 | Downregulates (4) | Activated |
| Fdps | FDPS | -2.521 | Regulates (2) | Affected |
| Csf1r | CSF1R | -2.582 | Upregulates (1) | Inhibited |
| Pole2 | POLE2 | -2.589 | Downregulates (1) | Activated |
| Phgdh | PHGDH | -2.666 | Downregulates (8) | Activated |
| Rfc3 | RFC3 | -2.747 | Downregulates (1) | Activated |
| Tyms | TYMS | -2.786 | Regulates (2) | Affected |
| Rad54b | RAD54B | -2.978 | Downregulates (1) | Activated |
| Pttg1 | PTTG1 | -3.218 | Upregulates (5) | Inhibited |
| Dbf4 | DBF4 | -3.344 | Downregulates (1) | Activated |
| Vrk1 | VRK1 | -3.378 | Downregulates (9) | Activated |
| Brca2 | BRCA2 | -3.516 | Downregulates (7) | Activated |
| Nkd1 | NKD1 | -3.543 | Downregulates (5) | Activated |
| Chek1 | CHEK1 | -3.62 | Downregulates (12) | Activated |
| Trib3 | TRIB3 | -3.666 | Downregulates (2) | Activated |
| Mis18a | MIS18A | -3.694 | Regulates (1) | Affected |
| Rbm3 | RBM3 | -3.782 | Upregulates (1) | Inhibited |
| Mcm7 | MCM7 | -3.785 | Downregulates (6) | Activated |
| Mcm6 | MCM6 | -3.944 | Downregulates (7) | Activated |
| Rrm1 | RRM1 | -4.187 | Regulates (2) | Affected |
| Thbd | THBD | -4.214 | Downregulates (3) | Activated |
| Pcna | PCNA | -4.28 | Upregulates (21) | Inhibited |
| Kif24 | KIF24 | -4.338 | Regulates (3) | Affected |
| Hjurp | HJURP | -4.565 | Regulates (1) | Affected |
| Pold1 | POLD1 | -4.941 | Downregulates (6) | Activated |
| Mad2l1 | MAD2L1 | -5.044 | Downregulates (4) | Activated |
| Rnf144b | RNF144B | -5.126 | Upregulates (2) | Inhibited |
| Mybl1 | MYBL1 | -5.463 | Upregulates (4) | Inhibited |
| H2afx | H2AX | -5.55 | Downregulates (5) | Activated |
| Mcm4 | MCM4 | -5.633 | Downregulates (3) | Activated |
| Kif23 | KIF23 | -5.685 | Downregulates (4) | Activated |
| Smc4 | SMC4 | -5.694 | Downregulates (1) | Activated |
| Ncapg | NCAPG | -5.887 | Downregulates (1) | Activated |
| E2f8 | E2F8 | -6.031 | Regulates (1) | Affected |
| Recql4 | RECQL4 | -6.145 | Downregulates (7) | Activated |
| Fignl1 | FIGNL1 | -6.17 | Regulates (1) | Affected |
| Psrc1 | PSRC1 | -6.377 | Upregulates (10) | Inhibited |
| Kifc1 | KIFC1 | -6.502 | Upregulates (2) | Inhibited |
| Aurka | AURKA | -6.58 | Downregulates (6) | Activated |
| Cdc25b | CDC25B | -6.868 | Downregulates (11) | Activated |
| Cenpf | CENPF | -6.892 | Downregulates (1) | Activated |
| Mis18bp1 | MIS18BP1 | -6.935 | Regulates (1) | Affected |
| Cdc20 | CDC20 | -7.001 | Downregulates (4) | Activated |
| Foxm1 | FOXM1 | -7.141 | Regulates (29) | Affected |
| Aspm | ASPM | -7.396 | Regulates (1) | Affected |
| Sema6a | SEMA6A | -7.496 | Upregulates (2) | Inhibited |
| Fanci | FANCI | -7.737 | Regulates (1) | Affected |
| Mcm3 | MCM3 | -7.988 | Downregulates (6) | Activated |
| Smc2 | SMC2 | -8.11 | Downregulates (3) | Activated |
| Cdc6 | CDC6 | -8.339 | Downregulates (2) | Activated |
| Dsn1 | DSN1 | -8.611 | Downregulates (1) | Activated |
| Ndc80 | NDC80 | -9.002 | Downregulates (2) | Activated |
| Fosl1 | FOSL1 | -9.149 | Upregulates (3) | Inhibited |
| Bub1 | BUB1 | -9.18 | Downregulates (5) | Activated |
| Matn4 | MATN4 | -9.918 | Upregulates (1) | Inhibited |
| Tpx2 | TPX2 | -10.013 | Downregulates (5) | Activated |
| Cdt1 | CDT1 | -10.052 | Downregulates (1) | Activated |
| Dut | DUT | -10.055 | Downregulates (7) | Activated |
| Kntc1 | KNTC1 | -10.567 | Regulates (1) | Affected |
| Exo1 | EXO1 | -10.586 | Regulates (2) | Affected |
| Spc25 | SPC25 | -10.758 | Upregulates (2) | Inhibited |
| Rad51ap1 | RAD51AP1 | -10.815 | Regulates (1) | Affected |
| Fam83d | FAM83D | -10.829 | Upregulates (2) | Inhibited |
| Cep55 | CEP55 | -11.071 | Downregulates (5) | Activated |
| Mcm5 | MCM5 | -11.537 | Downregulates (4) | Activated |
| Ttk | TTK | -11.898 | Downregulates (4) | Activated |
| Cdk1 | CDK1 | -12.307 | Downregulates (19) | Activated |
| Anln | ANLN | -12.876 | Regulates (2) | Affected |
| Asf1b | ASF1B | -12.989 | Regulates (1) | Affected |
| Birc5 | BIRC5 | -14.114 | Downregulates (42) | Activated |
| Plk1 | PLK1 | -14.209 | Downregulates (4) | Activated |
| Ns5atp9 | PCLAF | -14.39 | Downregulates (5) | Activated |
| Dlgap5 | DLGAP5 | -14.527 | Regulates (1) | Affected |
| Ube2t | UBE2T | -14.745 | Regulates (1) | Affected |
| Pbk | PBK | -15.037 | Downregulates (2) | Activated |
| Hmmr | HMMR | -15.447 | Downregulates (1) | Activated |
| Top2a | TOP2A | -15.721 | Downregulates (5) | Activated |
| Gtse1 | GTSE1 | -18.577 | Upregulates (5) | Inhibited |
| Mki67 | MKI67 | -18.625 | Downregulates (2) | Activated |
| Mybl2 | MYBL2 | -18.809 | Downregulates (1) | Activated |
| Prc1 | PRC1 | -20.321 | Downregulates (11) | Activated |
| Ccnb2 | CCNB2 | -21.046 | Regulates (4) | Affected |
| Ube2c | UBE2C | -23.513 | Downregulates (3) | Activated |
| Ccna2 | CCNA2 | -25.421 | Downregulates (5) | Activated |
| Aurkb | AURKB | -27.934 | Downregulates (5) | Activated |

© 2000-2020 QIAGEN. All rights reserved.

*For using IPA the Fold changes (FC) are converted to Expr Fold changes: for down-regulated genes Expr Fold change = -1/FC. Up-regulated genes are not affected and Expr Fold change = FC.

**Supplementary Table 5 Expression levels of E2F1 transcription regulators identified via upstream DNA binding sites (Elf5, Elk1 and SpiB). None was significantly deregulated over culture time or between different process setups. Elf5 and SpiB found to be not expressed (low expression levels are probably artefacts).**

| ID | Expression level [FPKM] | | | | | | | | | | | |
| --- | --- | --- | --- | --- | --- | --- | --- | --- | --- | --- | --- | --- |
|  | control | | | | uHSD v0.1 | | | | uHSD | | | |
|  | day 0 | day 1 | day 4 | day 11 | day 0 | day 1 | day 4 | day 11 | day 0 | day 1 | day 4 | day 11 |
| Elf5 | 0 | 0 | 0 | 0 | 379 | 330 | 0 | 0 | 0 | 0 | 0 | 811 |
| Elk1 | 404120 | 339326 | 440762 | 581136 | 424008 | 388713 | 565935 | 687921 | 440802 | 395743 | 582101 | 810130 |
| SpiB | 722 | 0 | 1485 | 0 | 0 | 645 | 904 | 0 | 0 | 0 | 0 | 0 |
